# NiMoO_4_ With High Oxidation States for Efficient Electrooxidation of Amines to Nitriles

**DOI:** 10.1002/EXP.20240327

**Published:** 2026-02-16

**Authors:** Hao Chen, Man Qiao, Zhixiang Yuan, Dazhi Yao, Dongdong Zhu, Ping Chen

**Affiliations:** ^1^ School of Materials Science and Engineering Anhui University Hefei Anhui China; ^2^ School of Chemistry and Materials Science Jiangsu Key Laboratory of New Energy Devices and Interface Science Nanjing University of Information Science and Technology Nanjing China; ^3^ School of Chemistry The University of New South Wales Sydney New South Wales Australia

**Keywords:** amine electrooxidation, electrocatalysis, NiMoO_4_, nitrile, oxidation state modulation

## Abstract

Electrochemical oxidation of amines to nitriles, with water as the O source, is a green and sustainable route to synthesize nitriles under mild conditions. However, developing highly efficient electrocatalysts for amine oxidation with large current densities is quite challenging. Herein, it is demonstrated that NiMoO_4_ with high oxidation states can efficiently catalyze benzylamine oxidation reaction (BAOR) to benzonitrile (BN) with a large anodic current density of 300.0 mA cm^−2^ at 1.47 V versus RHE. Impressively, NiMoO_4_ simultaneously achieves high BA conversion, BN selectivity, and faradaic efficiency of BN above 95%, together with a large BN yield of 0.366 mmol h^−1^ at 1.45 V versus RHE. Such excellent performance is also verified in a practical continuous‐flow membrane electrode assembly reactor. Electrochemical measurements and in situ techniques uncover that NiOOH is the catalytically active species for BAOR, while the generation of NiOOH is more facile and effective on NiMoO_4_ than on Ni(OH)_2_. Theoretical calculations reveal that Mo doping in NiOOH facilitates the adsorption of BA, and reduces the energy barriers for the subsequent dehydrogenation steps, leading to excellent BAOR performance.

## Graphical abstract

When water is used as an oxygen source, NiMoO_4_ electrocatalyst with high oxidation states can selectively and efficiently oxidize benzylamine (BA) to benzonitrile (BN) with high BA conversion, BN selectivity, and faradaic efficiency of BN above 95%.

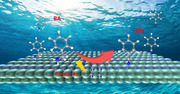

## 1 Introduction

Water electrolysis with renewable electricity has been regarded as a promising and sustainable way to produce green hydrogen [1, 2, 3, 4, 5]. In fact, by using water as an H or O source, electrochemical hydrogenation or oxidation of molecules offers a safe and efficient approach to synthesize hydrogenated or oxidized products under ambient conditions [6, 7, 8, 9]. Nitriles, with a −C≡N functional group, are an important class of organic compounds with widespread applications in pharmaceuticals, agrochemicals, and fine chemicals [10, 11, 12]. At present, industrial‐scale production of nitriles is dominated by hydrocyanation and ammoxidation. Hydrocyanation involves nucleophilic substitution reactions between alkenes and cyanide, which is highly toxic [13, 14]. Ammoxidation is more favorable, but it still requires the use of strong oxidants at high temperatures, resulting in safety concerns and high energy consumption [15]. Therefore, it is highly desirable to develop a green and energy‐efficient way to achieve mass production of nitriles under mild conditions. Recent advances reveal that electrochemical oxidation of amines (−CH_2_NH_2_) is an appealing way to synthesize nitriles, and such a dehydrogenation process is accomplished by using water as the green oxygen source [16]. The obtained nitrile product is easy to separate from the electrolyte owing to its hydrophobic property, thus avoiding the possible overoxidation of nitrile to achieve high selectivity [17]. Moreover, energy‐saving hydrogen production can be realized via using electrochemical oxidation of amines to replace the traditional oxygen evolution reaction at the anode of an electrolyzer, due to its intrinsic thermodynamic advantage [18].

Electrochemical oxidation of amines to nitriles is a complicated four‐electron transfer process with multiple steps, and it is imperative to develop highly active and selective electrocatalysts to promote this kinetically sluggish process [19]. Recently, some inspiring works have been reported in this emerging research area. Guo et al. found that CoSe_2_ with Se vacancies and Ni substitutions can efficiently oxidize benzylamine (BA) to benzonitrile (BN), and the current density for benzylamine oxidation reaction (BAOR) at 1.5 V (vs. reversible hydrogen electrode, RHE) is 120.0 mA cm^−2^ [20]. Zhai and co‐workers constructed vacancy‐rich Ni(OH)_2_ nanosheets for propylamine electrooxidation, and obtained anodic current density at 1.41 V versus RHE is 50.0 mA cm^−2^ [21]. Zhao et al. prepared Fe doped Ni_3_S_2_ electrocatalyst towards BAOR, and the current density at 1.5 V versus RHE is less than 90.0 mA cm^−2^ [22]. Chen and co‐workers reported that Ru‐modified Ni_2_P nanobelt electrode can selectively oxidize BA to BN, and a BAOR current density of 100.0 mA cm^−2^ was attained at 1.4 V versus RHE [14]. In spite of these significant achievements, there is still much room for the catalyst performance improvement. For instance, the currently reported electrocatalysts for amine electrooxidation only show moderate catalytic activity, as the anodic current densities are generally below 300 mA cm^−2^ at 1.5 V versus RHE [23, 24, 25]. Moreover, the underlying reaction mechanism of amine electrooxidation needs to be further explored.

Herein, NiMoO_4_ catalyst with high oxidation states was prepared on nickel foam (NF) for BAOR, which affords a large anodic current density of 300.0 mA cm^−2^ at 1.47 V versus RHE. The as‐fabricated NiMoO_4_ shows excellent BAOR performance with high BA conversion, BN selectivity, and faradaic efficiency (FE) of BN above 95% at 1.45 V versus RHE. Moreover, the BN yield of NiMoO_4_ at 1.45 V versus RHE is 0.366 mmol h^−1^, much higher than that of Ni(OH)_2_ sample (0.156 mmol h^−1^). In addition, the NiMoO_4_ catalyst can stably operate for 30 cycles in a continuous‐flow membrane electrode assembly (MEA) electrolyzer. In situ characterizations uncover that NiMoO_4_ guarantees fast and efficient generation of NiOOH, which is regarded as a catalytically active species for BAOR. Theoretical calculations further demonstrate that the introduction of Mo in NiOOH promotes the adsorption of BA molecules on the catalyst surface and reduces the energy barriers for the following dehydrogenation steps from BA to BN, thus contributing to outstanding BAOR performance.

## 2 Results and Discussion

**FIGURE 1:**
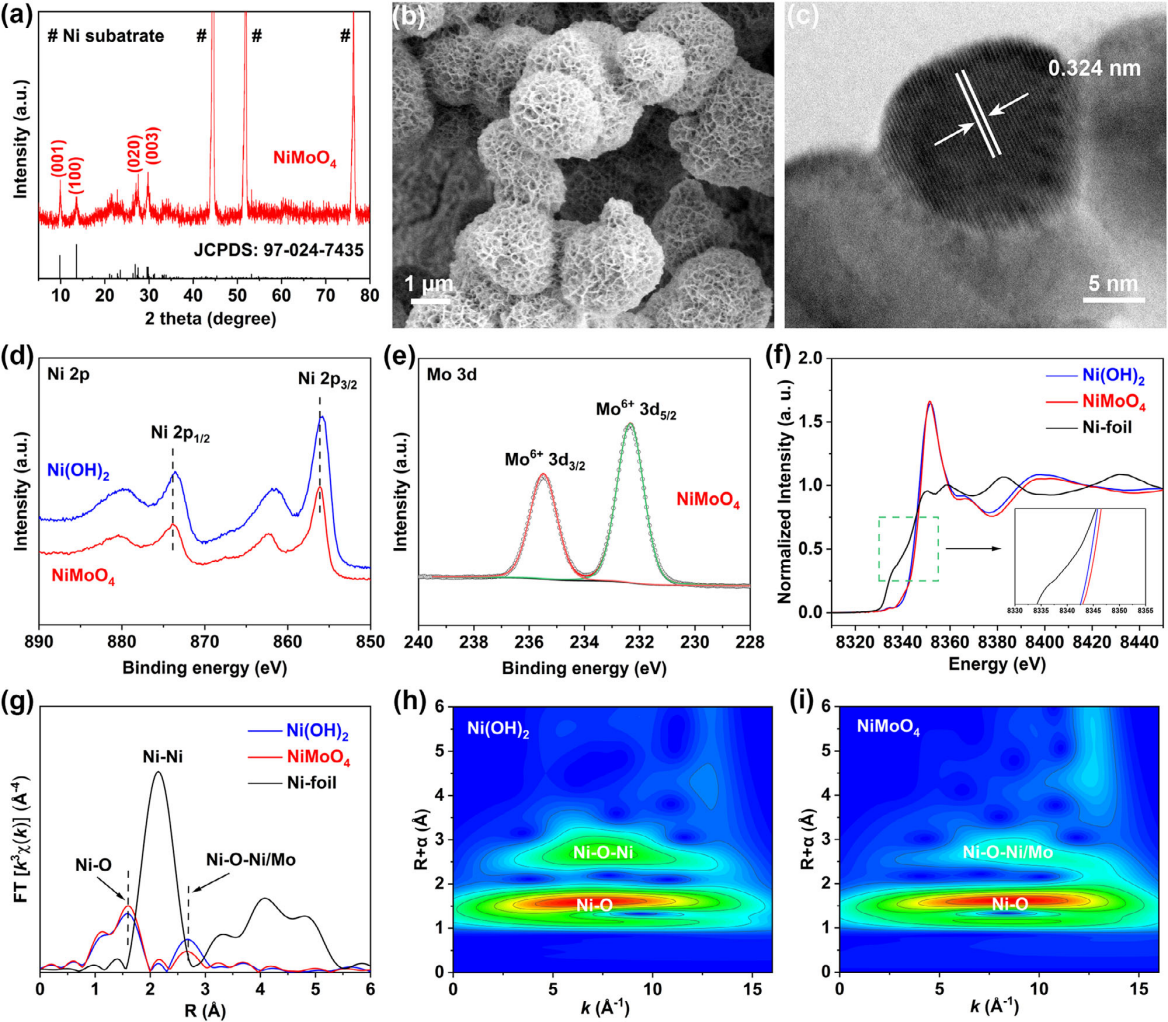
(a) XRD pattern of NiMoO_4_. (b) SEM image of NiMoO_4_. (c) HRTEM image of NiMoO_4_. (d) XPS Ni 2p spectra of Ni(OH)_2_ and NiMoO_4_. (e) XPS Mo 3d spectrum of NiMoO_4_. (f) Ni K‐edge XANES spectra of Ni(OH)_2_, NiMoO_4_, and Ni foil. (g) Ni K‐edge FT‐EXAFS spectra of Ni(OH)_2_, NiMoO_4_, and Ni foil. WT‐EXAFS contour plots of (h) Ni(OH)_2_, and (i) NiMoO_4_.

The NiMoO_4_ catalyst was synthesized on NF by a facile hydrothermal method (see Experimental section for details, and Figure S1, Supporting Information). As shown in Figure 1a, except for three sharp peaks stemmed from the NF substrate, the X‐ray diffraction (XRD) pattern of the obtained sample is well indexed to NiMoO_4_·0.7H_2_O (JCPDS No. 97‐024‐7435) [26, 27]. Therefore, for simplicity, the obtained catalyst is named NiMoO_4_. The scanning electron microscopy (SEM) images (Figure 1b and Figure S2, Supporting Information) display that the NiMoO_4_ microspheres are covered on the NF substrate, and nanosheets can be observed on the surface of these NiMoO_4_ microspheres. However, when sodium laurylsulfonate was not used during the hydrothermal process, the morphology of the obtained NiMoO_4_ sample completely changed, with dense and irregular nanorods generated on the NF substrate (Figure S3, Supporting Information). The high‐resolution transmission electron microscopy (HRTEM) image in Figure 1c exhibits clear lattice fringes with an interplanar distance of 0.324 nm, which corresponds well to the (020) plane of NiMoO_4_·0.7H_2_O, again confirming the successful preparation of NiMoO_4_. The high‐angle annular dark‐field scanning TEM (HAADF‐STEM) image, and corresponding elemental mapping results of NiMoO_4_ (Figure S4, Supporting Information) reveal the uniform distribution of Ni, Mo, and O elements. For comparison, a typical nickel‐based catalyst, Ni(OH)_2_ was also prepared (Figure S5, Supporting Information). The N_2_ adsorption–desorption isotherms (Figure S6, Supporting Information) show that the Brunauer–Emmett–Teller (BET) surface area of NiMoO_4_ (288.37 m^2^ g^−1^) exceeds that of Ni(OH)_2_ (111.54 m^2^ g^−1^). X‐ray photoelectron spectroscopy (XPS) survey spectrum of NiMoO_4_ verifies the coexistence of Ni, Mo, and O elements (Figure S7, Supporting Information). Notably, as displayed in Figure 1d, compared to Ni(OH)_2_ sample, all the Ni 2p peaks of NiMoO_4_ shift noticeably to higher binding energy regions, suggesting a higher oxidation state of Ni cations in NiMoO_4_. The fitted Ni 2p spectra of NiMoO_4_ and Ni(OH)_2_ (Figure S8, Supporting Information) also confirm that NiMoO_4_ has a higher content of Ni^3+^ species than that of Ni(OH)_2_. The Mo 3d spectrum of NiMoO_4_ in Figure 1e shows two main peaks positioned at 235.5 and 232.4 eV, which correspond well to Mo^6+^ 3d_3/2_, and Mo^6+^ 3d_5/2_, respectively [28, 29]. The XPS O 1s spectrum of NiMoO_4_ (Figure S9, Supporting Information) exhibits two peaks at 530.7, and 533.0 eV, well assigned to metal–O (M‐O) bond, and O−H bond from surface water [30]. X‐ray absorption spectroscopy (XAS) was further applied to investigate the chemical state and local coordination environment of the samples. The Ni K‐edge X‐ray absorption near‐edge structure (XANES) spectra of NiMoO_4_, Ni(OH)_2_, and Ni foil are presented in Figure 1f. Compared to Ni(OH)_2_, the absorption edge of NiMoO_4_ moves to higher energy regions, confirming the higher oxidation state of Ni cations in NiMoO_4_, which aligns with previous XPS results. Fourier transform *k^3^*‐weighted extended X‐ray absorption fine structure (FT‐EXAFS) spectra of the samples are displayed in Figure 1g. Notably, for NiMoO_4_, the prominent peak at ≈1.6 Å is attributed to Ni‐O coordination in the first shell, while the peak appeared at ≈2.7 Å is assigned to Ni‐O‐Ni/Mo (Ni‐Ni/Mo) coordination in the second shell [31, 32, 33]. The presence of Ni‐O‐Ni/Mo in NiMoO_4_ is further corroborated by wavelet transform (WT)‐EXAFS analysis. The WT contour plots of Ni(OH)_2_ in Figure 1h exhibit a sharp zone (*R* = 2.7 Å and *k* = 7.5 Å^−1^), which is attributed to the Ni‐O‐Ni coordination. In contrast, as displayed in Figure 1i, NiMoO_4_ has a distinct k value greater than 7.5 Å^−1^, indicating the presence of Ni‐O‐Ni/Mo rather than Ni‐O‐Ni in the second shell for NiMoO_4_. In conclusion, all the results above clearly demonstrate that NiMoO_4_ with high oxidation states was successfully prepared.

**FIGURE 2:**
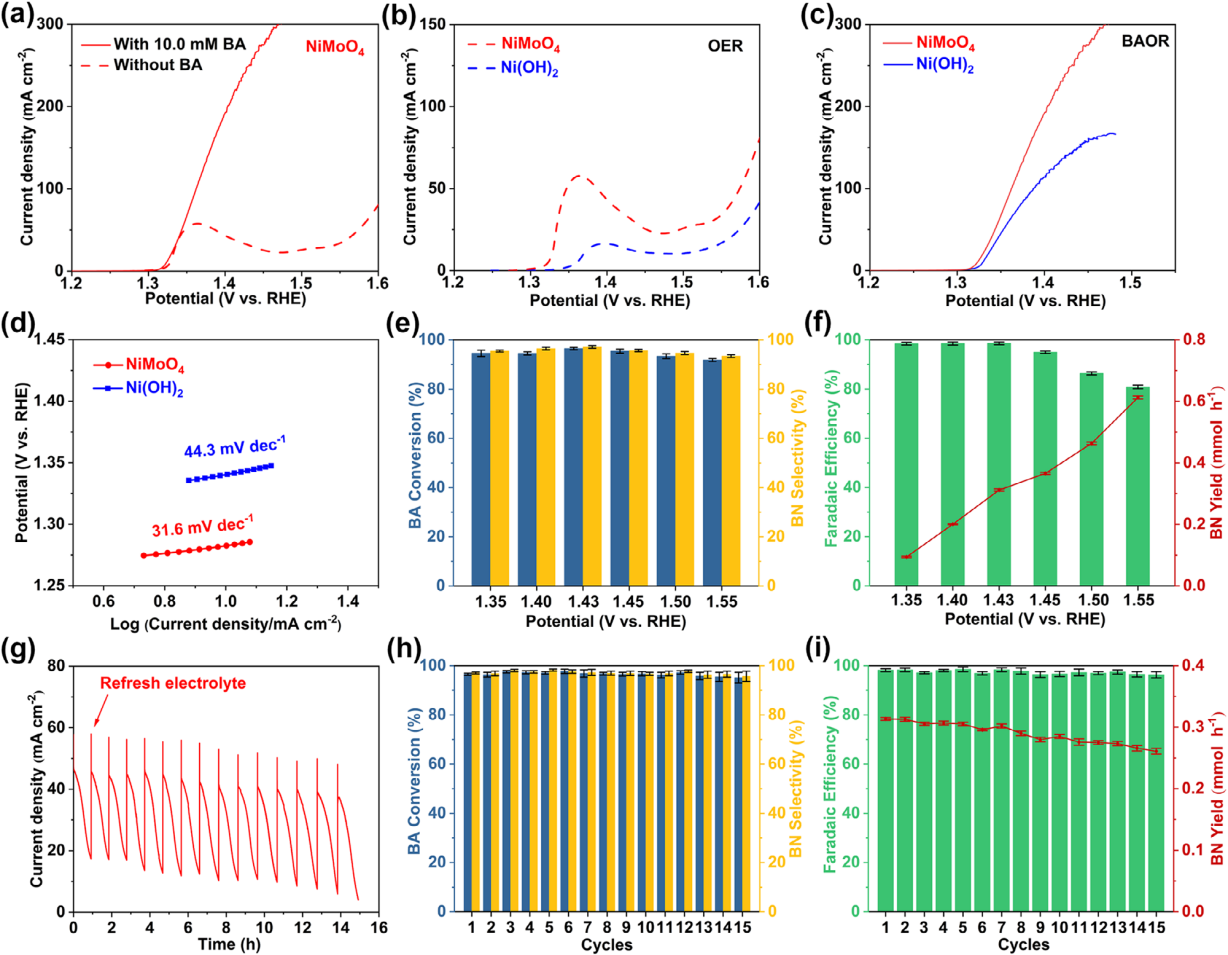
(a) LSV curves of NiMoO_4_ in 1.0 M KOH with or without 10.0 mM BA. (b) LSV curves of NiMoO_4_ and Ni(OH)_2_ in 1.0 M KOH. (c) LSV curves of NiMoO_4_ and Ni(OH)_2_ in 1.0 M KOH with 10.0 mM BA. (d) Tafel plots of NiMoO_4_ and Ni(OH)_2_. (e) BA conversion, and BN selectivity of NiMoO_4_ at various potentials. (f) FE of BN and BN yield of NiMoO_4_ at various potentials. (g) The successive chronoamperometry test of NiMoO_4_ at 1.43 V versus RHE for 15 cycles. (h) BA conversion, and BN selectivity of NiMoO_4_ for 15 cycles. (i) FE of BN, and BN yield of NiMoO_4_ for 15 cycles.

To evaluate the catalytic performance of the as‐synthesized NiMoO_4_, a series of electrochemical measurements was carried out using a three‐electrode configuration in a two‐chambered H‐type electrolytic cell first. Figure 2a shows the linear sweep voltammetry (LSV) curves of NiMoO_4_ in different electrolytes. It is worth noting that 90%*iR compensation is applied to the LSV curves. When performed in 1.0 M KOH, there is a significant oxidation peak at around 1.36 V versus RHE, which is attributed to the oxidation of Ni^2+^ to Ni^3+^ species [34, 35]. Afterward, OER starts with a high onset potential of 1.47 V versus RHE. After the addition of 10.0 mM BA, NiMoO_4_ exhibits a low onset potential of 1.3 V versus RHE toward BAOR, together with greatly enhanced anodic current densities. Herein, such an onset potential advantage means that by choosing a suitable applied potential, the competing OER can be completely avoided during BAOR. It is generally accepted that for Ni‐based catalysts, the in situ formed NiOOH species provides catalytically active sites for amine oxidation reactions, including BAOR [36, 37]. Therefore, fast and efficient generation of NiOOH species is critical for achieving outstanding BAOR performance. As exhibited in Figure 2b, NiMoO_4_ has a much lower onset potential for Ni^2+^ oxidation to Ni^3+^ than that of Ni(OH)_2_, accompanied by a greatly increased oxidation peak area, indicating that the generation of active NiOOH species is more facile on NiMoO_4_. Not surprisingly, compared to Ni(OH)_2_, NiMoO_4_ shows a lower onset potential and higher current densities toward BAOR (Figure 2c). For instance, at 1.47 V versus RHE, NiMoO_4_ achieves an industrial current density of 300.0 mA cm^−2^, while the current density for Ni(OH)_2_ is only 164.7 mA cm^−2^. The bare NF was also tested for BAOR, while its poor catalytic performance confirms that the intrinsic catalytic activity for BAOR originates from the NiMoO_4_ catalyst, rather than the NF substrate (Figure S10, Supporting Information). NiMoO_4_ also possesses much higher current densities toward BAOR than commercial RuO_2_. In fact, such exceptional BAOR performance of NiMoO_4_ has rarely been reported before (Table S1, Supporting Information). Moreover, NiMoO_4_ has a larger double‐layer capacitance (*C*_dl_) value than that of Ni(OH)_2_ (Figure S11, Supporting Information), suggesting that NiMoO_4_ with a larger electrochemically active surface area can provide more catalytically active sites toward BAOR, contributing to its improved BAOR activity. In Figure 2d, the Tafel slope of NiMoO_4_ is smaller (31.6 mV dec^−1^) than that of Ni(OH)_2_ (44.3 mV dec^−1^), implying a more favorable reaction kinetics of NiMoO_4_ toward BAOR. To investigate the potential dependence of BAOR performance, a chronoamperometry test with the total passing charge of 115.6 C was carried out at different potentials in 1.0 M KOH containing 10.0 mM BA (Figures S12 and S13, Supporting Information). The oil droplets can be clearly observed on the surface of the electrolyte after BAOR (Figure S14, Supporting Information). The obtained liquid products were measured by a gas chromatograph (GC) with calibration curves (Figure S15, Supporting Information). After BAOR with a 30 C passing charge, BN appeared as the product, while the amount of BA substrate reduced (Figure S16, Supporting Information). When 115.6 C was consumed for BAOR, almost all the BA substrate was converted to BN. As shown in Figure 2e, NiMoO_4_ achieves high BA conversion and BN selectivity above 90% at a wide potential window from 1.35 to 1.55 V versus RHE. The FE of the desired BN is more than 95% when the potential is no higher than 1.45 V versus RHE in Figure 2f. However, due to the competing OER, the FE of BN reduces significantly when the potential is above 1.45 V versus RHE (Figure S17, Supporting Information). Meanwhile, the BN yield increases with the applied more positive potential, and a large BN yield of 0.366 mmol h^−1^ is attained at 1.45 V versus RHE. In contrast, Ni(OH)_2_ sample achieves reduced FEs of BN, and the BN yield at 1.45 V versus RHE is only 0.156 mmol h^−1^ (Figure S18, Supporting Information). The durability performance of NiMoO_4_ for BAOR was evaluated by a continuous cycling test for 15 cycles at the potential of 1.43 V versus RHE, and the passing charge for each cycle is 115.6 C (Figure 2g). As depicted in Figure 2h,i, though the reaction rate of BAOR is slightly influenced, NiMoO_4_ presents good BAOR stability with no obvious attenuation of BA conversion, BN selectivity, or FE of BN during the 15‐cycle test. The BAOR stability performance of Ni(OH)_2_ was also investigated (Figure S19 and S20, Supporting Information), and the BN yield on Ni(OH)_2_ is much lower than that of NiMoO_4_ at each cycle. To explore the BAOR performance of NiMoO_4_ in low alkaline medium, we carried out the test in 0.1 M KOH/0.5 M Na_2_SO_4_ with 10.0 mM BA (Figure S21, Supporting Information). The obtained results clearly show that NiMoO_4_ is still highly efficient for BAOR in low alkaline medium. High BA conversion, BN selectivity, and FE of BN are achieved at a wide potential window from 1.45 to 1.65 V versus RHE. To demonstrate the general applicability of NiMoO_4_ for amine oxidation to nitrile, cyclohexenylethylamine was chosen as the substrate. The LSV curves show that NiMoO_4_ is highly active for cyclohexenylethylamine oxidation (Figure S22, Supporting Information). When the applied potential is no higher than 1.45 V versus RHE, large amine conversion, nitrile selectivity, and FE of nitrile above 90% are attained, highlighting the great potential of NiMoO_4_ electrode for other amine oxidation reactions.

**FIGURE 3:**
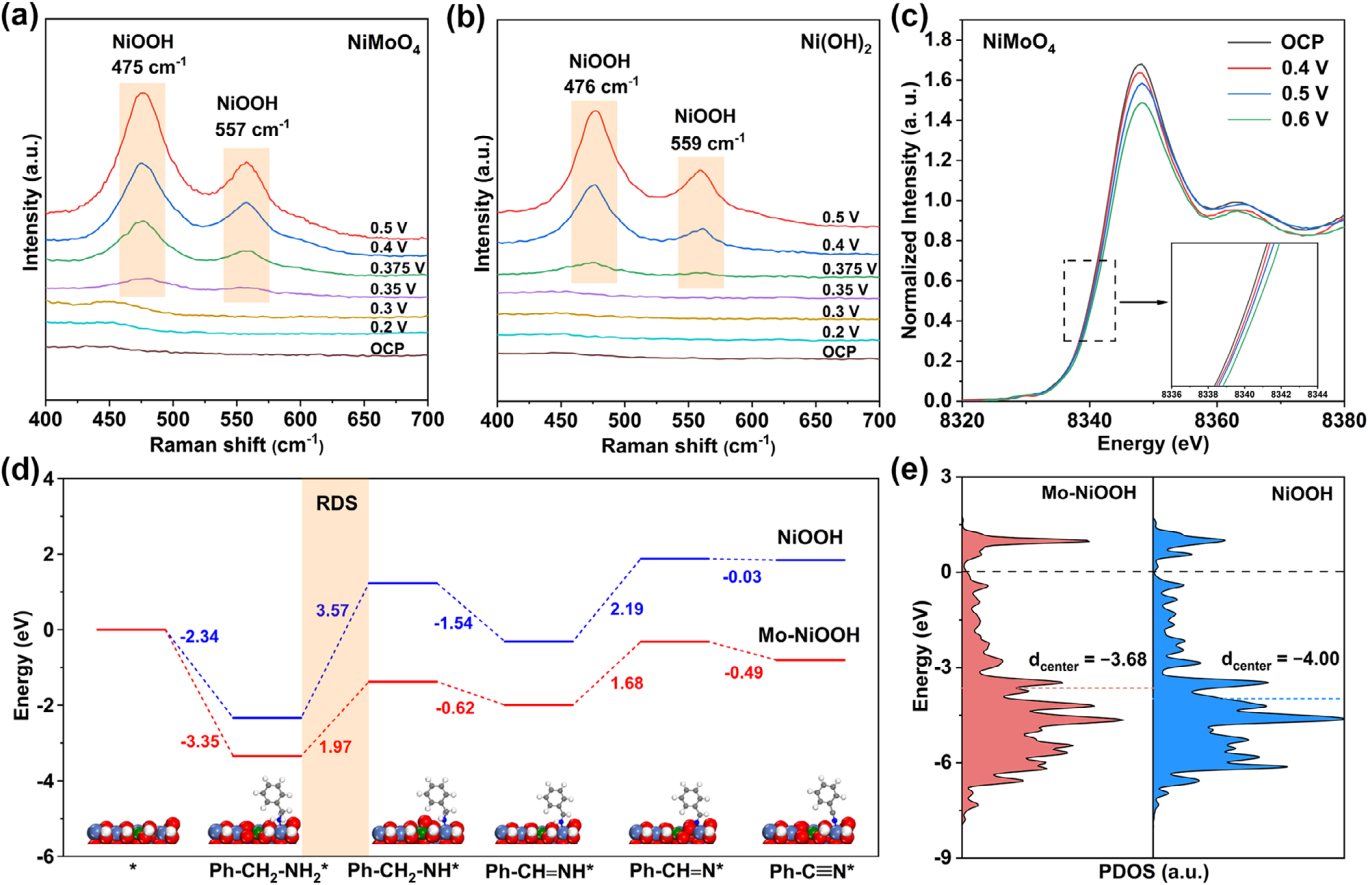
(a) Potential‐dependent in situ Raman spectra of NiMoO_4_ electrode collected in 1.0 M KOH with 10.0 mM BA. (b) Potential‐dependent in situ Raman spectra of Ni(OH)_2_ electrode collected in 1.0 M KOH with 10.0 mM BA. (c) In situ Ni K‐edge XANES spectra of NiMoO_4_ electrode during the BAOR process. (d) Free energy diagrams for BAOR and corresponding intermediate adsorption on Mo‐NiOOH and NiOOH. (e) PDOS of Mo‐NiOOH and NiOOH.

To probe the structure evolution of the catalysts during BAOR, in situ techniques were applied. As shown in Figure 3a, in situ Raman spectra of NiMoO_4_ show that when the potential increases to 0.35 V versus Ag/AgCl, two distinct peaks at 475 and 557 cm^−1^ appear, which are well assigned to *β*‐NiOOH [38]. However, for Ni(OH)_2_, NiOOH can only be observed when the potential is no less than 0.375 V versus Ag/AgCl (Figure 3b), giving strong evidence that the generation of active NiOOH species is more facile on NiMoO_4_. Moreover, when the reaction time increases from 30 to 600 s (Figure S23, Supporting Information), the Raman peak intensity is gradually enhanced, revealing the formation of more NiOOH species. In situ Ni K‐edge XANES spectra in Figure 3c uncover that BAOR on NiMoO_4_ is closely associated with the oxidation state change of Ni cations. In detail, the oxidation state of Ni increases with the applied more positive potential, indicating that the in situ formed high‐valence Ni species provides catalytically active sites for BAOR. Density functional theory (DFT) calculations were further carried out to elucidate the underlying mechanism of BAOR. Based on the experimental findings that the in situ generated NiOOH species is the real catalytically active species for BAOR, the constructed structural models for NiMoO_4_ and Ni(OH)_2_ catalysts are Mo‐NiOOH and NiOOH, respectively (see Experimental Section for computational details, and Figure S24, Supporting Information). The reaction pathway and energy diagrams for BAOR to BN on NiOOH and Mo‐NiOOH models are presented in Figure 3d. The BAOR initials with the adsorption of BA, and the adsorption energy (Ph−CH_2_−NH_2_*) is calculated to be −2.34 and −3.35 eV for NiOOH and Mo‐NiOOH, respectively. The relatively strong adsorption of BA on Mo‐NiOOH is desirable for BA activation for the subsequent oxidation reaction. Afterward, BAOR proceeds with continuous dehydrogenation steps: Ph−CH_2_−NH_2_* → Ph−CH_2_−NH* → Ph−CH = NH* → Ph−CH = N* → Ph−C≡N*. The rate‐determining step (RDS) of the entire BAOR is the first dehydrogenation step, which forms the Ph−CH_2_−NH* intermediate. The energy barrier of this RDS is 1.97 eV for Mo‐NiOOH, which is much smaller than that of NiOOH (3.57 eV), demonstrating that Mo incorporation promotes BAOR. The enhanced adsorption of BA can be reflected by the electronic interaction between the catalyst and BA [39]. Charge density difference calculation (Figure S24, Supporting Information) shows that Mo‐NiOOH gains 0.34 |e^−^| from the BA molecule, while only 0.15 |e^−^| transfers to NiOOH. It is well suspected that highly oxidized Ni in Mo‐NiOOH is electron‐deficient, which is beneficial for the adsorption of BA by attracting the lone pair electrons from its amine group (−NH_2_) [21]. Moreover, projected density of state (PDOS) was used to reflect the change of the d‐band center (*ɛ*_d_). As shown in Figure 3e, the *ɛ*_d_ of Mo‐NiOOH (−3.68 eV) is closer to the Fermi level (*E*_f_) than that of NiOOH (−4.00 eV), indicating that Mo doping in NiOOH leads to the upshift of *ɛ*_d_, which raises the antibonding level, contributing to enhanced BA adsorption [40, 41]. Overall, DFT results indicate that the introduction of Mo in NiOOH is favorable for the chemical adsorption of BA, and subsequent dehydrogenation steps from BA to BN. Moreover, NiMoO_4_ catalyst can guarantee fast and efficient generation of active Mo‐NiOOH species, which is responsible for the excellent BAOR results.

**FIGURE 4:**
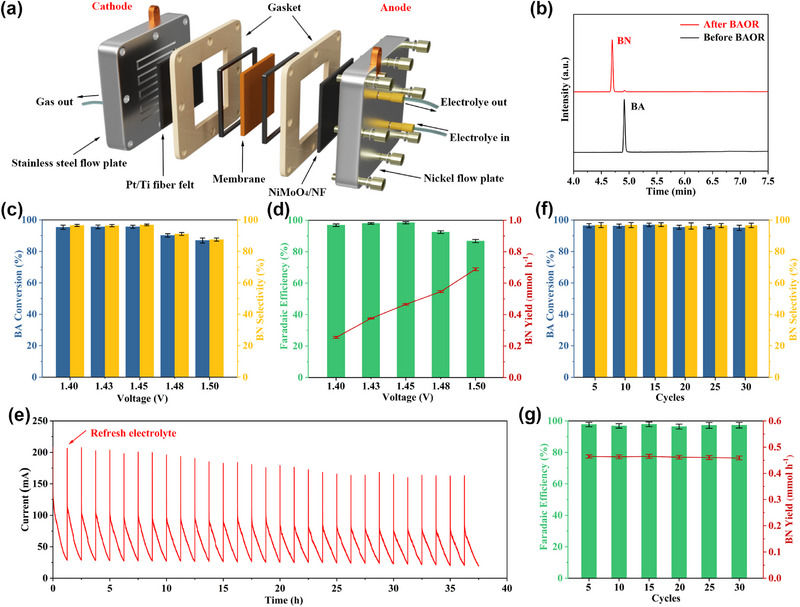
(a) Schematic diagram of a continuous‐flow MEA reactor. (b) Detection of the anode product after BAOR. (c) BA conversion, and BN selectivity at different cell voltages. (d) FE of BN, and BN yield at different cell voltages. (e) Stability test with a constant cell voltage of 1.45 V for 30 cycles. (f) BA conversion, and BN selectivity during the stability test. (g) FE of BN, and BN yield during the stability test.

Inspired by the remarkable BAOR performance of NiMoO_4_ catalyst in the three‐electrode system, we further evaluated the performance of NiMoO_4_ in a more practical scenario. Specifically, as shown in Figure 4a, a continuous‐flow MEA reactor using NiMoO_4_/NF as the anode, and commercial Pt/Ti fiber felt (Figure S25, Supporting Information) as the cathode was constructed for two‐electrode tests. Peristaltic pumps were used to drive the electrolyte circulation for supplying raw materials to the electrode surface and quickly removing the products (Figure S26, Supporting Information) [42]. As illustrated in Figure S27, Supporting Information, water is reduced at the cathode to produce hydrogen, while BA is oxidized at the anode. GC was used to detect the liquid product at the anode, and discovered that the product of BA oxidation in this continuous‐flow reactor is still BN (Figure 4b). High BA conversion, BN selectivity, and FE of BN beyond 90% are attained under the wide voltage range from 1.4 to 1.48 V (Figure 4c,d). Moreover, a large BN yield of 0.689 mmol h^−1^ was obtained at 1.5 V. The durability behavior of NiMoO_4_ was investigated by a chronoamperometry test at 1.45 V for 30 cycles (Figure 4e). Impressively, high BA conversion, BN selectivity, and FE of BN above 95% are well retained during the 30 consecutive cycles (Figure 4f,g). In addition, a quite stable and large BN yield of around 0.46 mmol h^−1^ was achieved during the whole stability test. To uncover the possible change of the catalyst during BAOR, a series of characterizations was performed after the cycling test. XRD patterns show that, besides NiMoO_4_, NiOOH emerges after the BAOR cycling test (Figure S28a, Supporting Information). Moreover, characteristic peaks assigned to NiOOH species appear in the Raman spectrum of NiMoO_4_ after the cycling test, again confirming the generation of NiOOH (Figure S28b, Supporting Information). Ni K‐edge XANES spectra (Figure S28c, Supporting Information) also reveal the formation of Ni species with higher oxidation states, in agreement with previous results. SEM image in Figure S28d, Supporting Information, indicates that the original microsphere morphology of NiMoO_4_ is well retained. Thus, it is well suspected that the relatively stable in‐situ formed NiOOH/NiMoO_4_ structure and morphology contribute to the excellent stability performance.

## 3 Conclusions

In summary, the NiMoO_4_ catalyst was successfully prepared by a typical hydrothermal method. XPS and XAS results confirm the higher oxidation states of Ni cations in NiMoO_4_, compared to Ni(OH)_2_. The as‐synthesized NiMoO_4_ with high oxidation states shows exceptional performance towards BAOR, and the current density at 1.47 V versus RHE is 300.0 mA cm^−2^. Specifically, with the applied potential of 1.45 V versus RHE, NiMoO_4_ achieves high BA conversion, BN selectivity, and FE of BN above 95%, while its BN yield is more than twice higher than that of Ni(OH)_2_. Moreover, when tested as the anode in a continuous‐flow MEA reactor, NiMoO_4_ displays outstanding BAOR stability with large BN yields of about 0.46 mmol h^−1^ for 30 cycles. Electrochemical method, in situ Raman, and in situ XAS demonstrate that NiOOH is the real active species for BAOR, and its generation is more facile and effective on NiMoO_4_. DFT calculations clearly show that Mo incorporation in NiOOH facilitates the BA adsorption, and greatly reduces the energy barrier of the rate‐determining step (formation of Ph−CH_2_−NH* intermediate) from 3.57 to 1.97 eV, thus contributing to the excellent BAOR performance. This work not only provides a highly efficient and stable electrocatalyst for BAOR but also highlights the importance of oxidation state modulation in the electrocatalyst design for organic oxidation reactions.

## Conflicts of Interest

The authors declare no conflicts of interest.

## Supporting information




**Supporting File 1**: exp270133‐sup‐0001‐SuppMat.docx.

## Data Availability

The data that support the findings of this study are available from the corresponding author upon reasonable request.
