# Supplementary material for: NiMoO4 With High Oxidation States for Efficient Electrooxidation of Amines to Nitriles
Source: Exploration (Beijing). 2026 Feb 16;6(1):20240327. doi: 10.1002/EXP.20240327 (PMC12970222; doi:10.1002/EXP.20240327)
Supplement: Supplementary file 1 — Supporting File 1: exp270133‐sup‐0001‐SuppMat.docx. [file EXP2-6-20240327-s001.docx]

**Supplementary Information**

NiMoO_4_ with high oxidation states for efficient electrooxidation of amines to nitriles

*Hao Chen*^+^ *^a^, Man Qiao*^+^ *^b^, Zhixiang Yuan ^a^, Dazhi Yao ^c^, Dongdong Zhu* ^b^ and Ping Chen* ^a^*

^a^ School of Materials Science and Engineering, Anhui University, Hefei, Anhui, China

E-mail: chenping@ahu.edu.cn

^b^ School of Chemistry and Materials Science, Jiangsu Key Laboratory of New Energy Devices and Interface Science, Nanjing University of Information Science and Technology, Nanjing, China

E-mail: dd.zhu@nuist.edu.cn

^c^ School of Chemistry, The University of New South Wales, Sydney, NSW 2052, Australia

^+^ These authors contributed equally to this work.

1. **Experimental Section**
   1. **Chemicals**

Nickel (Ⅱ) nitrate hexahydrate (Ni(NO_3_)_2_·6H_2_O, ≥98%), potassium hydroxide (KOH, ≥85%), urea (CO(NH_2_)_2_, ≥99%), and ethanol (CH_3_CH_2_OH, ≥99.7%) were purchased from Sinopharm reagent Co., Ltd. Sodium molybdate dihydrate (Na_2_MoO_4_·2H_2_O, 99%), benzylamine (BA, C_7_H_9_N, 99%), and benzonitrile (BN, C_7_H_5_N, ≥99.5%) were purchased from Macklin Biochemical Co., Ltd. Sodium laurylsulfonate (SLS, C_12_H_25_NaO_3_S, ≥98%) was purchased from Aladdin Chemical Reagent Co., Ltd. All of these reagents were of analytical grade, and directly used without further purification. Deionized (DI) water was used in all experiments.

- 1. **Synthesis of NiMoO_4_**

A piece of nickel foam (NF, 3.5 cm×2 cm) was ultrasonicated with acetone, water, and 3.0 M HCl aqueous solutions for 15 min, respectively. Then, the NF was rinsed with DI water. Lastly, the NF was dried in vacuum at 60 ^o^C. The NiMoO_4_ grown on NF was prepared by a simple hydrothermal method. Firstly, 2.8 mmol Ni(NO_3_)_2_·6H_2_O, 1.4 mmol Na_2_MoO_4_·2H_2_O, and 0.2 g C_12_H_25_NaO_3_S were dissolved in 60 mL of DI water, followed by ultrasonication treatment for 20 min to form a homogeneous solution. Then the resulting solution was transferred to a 100 mL Teflon-lined stainless steel autoclave containing a piece of clean NF. The autoclave was first heated at 70 ^o^C for 1.0 h, and then heated at 100 ^o^C for 6.0 h. After cooling to room temperature naturally, the NF was taken out from the Teflon-lined autoclave, washed with DI water several times, and dried in a vacuum oven at 60 ^o^C for 12 h.

- 1. **Synthesis of Ni(OH)_2_**

Ni(OH)_2_ on NF was also prepared by the hydrothermal method. Firstly, 4.2 mmol Ni(NO_3_)_2_·6H_2_O and 9 mmol urea were dissolved in 60 mL of DI water, followed by ultrasonication treatment for 20 min to form a homogeneous solution. Then the obtained solution was transferred to a 100 mL Teflon-lined stainless steel autoclave containing a piece of clean NF. The autoclave was heated at 100 ^o^C for 6 h. After naturally cooling to room temperature, the NF was taken out from the Teflon-lined autoclave, washed with DI water several times, and dried in a vacuum oven at 60 ^o^C for 12 h.

- 1. **Physical Characterizations**

The XRD pattern was obtained by using a diffractometer (Rigaku SmartLab 9KW with Cu Kα radiation, λ = 0.15406 nm) from 5 to 80° at a rate of 10° min^–1^. The scanning electron microscopy (SEM) images were obtained from a Hitachi S-4800 scanning electron microscope (3 kV). The transmission electron microscopy (TEM), and higher-resolution transmission electron microscopy (HRTEM) results were obtained on JEM-2100F (JEOL, Japan). X-ray photoelectron spectroscopy (XPS) data was obtained using a Thermo ESCALAB 250Xi X-ray photoelectron spectrometer (Thermo ESCALAB 250Xi). All the peaks were calibrated with C 1s spectrum at binding energy of 284.8 eV. The X-ray absorption spectra (XAS) were conducted on TableXAFS-500 (Specreation Instruments Co., Ltd., China).

- 1. **Electrochemical measurements**

Electrochemical tests were first carried out with a three-electrode system in an H-type electrochemical cell separated by an anion exchange membrane (FAA-3-PK130, Fumasep) on an electrochemical workstation (CHI 660E, CH instrument, China). The self-supported NiMoO_4_/NF with a surface area of 1.75 cm^2^ was directly used as the working electrode, while the Hg/HgO (1.0 M KOH) electrode and platinum plate were used as the reference and counter electrodes, respectively. All potentials measured were calibrated to reversible hydrogen electrode (RHE) using the following equation: *E*_RHE_ = *E*_Hg/HgO_ + 0.098 + 0.0591×pH. The electrolytes used for BAOR and OER are 1.0 M KOH solution containing 10 mM BA, and 1.0 M KOH, respectively. Linear sweep voltammetry (LSV) measurements were conducted at a scan rate of 5 mV s^−1^. The LSV curves were corrected with 90%*iR compensation. Cyclic voltammogram (CV) method was employed to estimate the double-layer capacitance (C_dl_) of the samples in a small non-faradaic region from 0 to 0.1 V *vs.* Hg/HgO at different scan rates. To investigate the potential dependence of BAOR performance, chronoamperometry test with the total passing charge of 115.6 C was carried out at different potentials in 1.0 M KOH containing 10.0 mM BA. The BAOR stability performance was evaluated by chronoamperometry test at 1.43 V *vs.* RHE for 15 cycles. The passing charge for each cycle is 115.6 C, and the electrolyte is refreshed after each cycle. For comparison, Ni(OH)_2_/NF with a surface area of 1.75 cm^2^ was used for BAOR. Self-supported NiMoO_4_/NF with a surface area of 1.75 cm^2^ was also used as the working electrode for BAOR test in 0.1 M KOH/0.5 M Na_2_SO_4_ with 10.0 mM BA, and amine oxidation test in 1.0 M KOH with 10.0 mM cyclohexenylethylamine.

For the continuous-flow Membrane Electrode Assembly (MEA) reactor, NiMoO_4_/NF (4.0 cm^2^) and Pt/Ti fiber felt (4.0 cm^2^) were employed as the anode and cathode, respectively. The electrolytes used for the anode and cathode were 1.0 M KOH with 10 mM BA, and 1.0 M KOH, respectively. The membrane used in MEA is FAA-3-PK130 (Fumasep). Peristaltic pumps were used to drive the electrolyte circulation. The voltage dependence of BAOR performance on NiMoO_4_ was investigated by chronoamperometry test at different cell voltages in 1.0 M KOH containing 10.0 mM BA, and each voltage test lasts for 1.25 h. The MEA’s stability performance for BAOR was evaluated by chronoamperometry test at 1.45 V for 30 cycles. All electrochemical measurements were conducted at ambient conditions.

- 1. **Product Quantification**

To analyze the liquid products of BAOR, 30 mL of electrolyte solution was extracted using ethyl acetate (60 mL) after each chronoamperometry test. The extracted liquid products were analyzed quantitatively by CEAULIGHT GC-7920 equipped with thermal conductivity detector (TCD) and flame ionization detector (FID). The injection temperature was set at 250 ^o^C. Nitrogen was used as the carrier gas at 1.5 mL min^-1^. The theoretical amount of charge transferred during the selective oxidation of all BA in the anode chamber to BN is calculated by the following equation:

$$\text{(}\text{10}\text{ }\text{× }\text{10}^{\text{-3}}\text{mol }\text{L}^{\text{-1}}\text{ × 30 × }\text{10}^{\text{-3}}\text{L) × 4 ×(6.02×}\text{10}^{\text{23}}\text{mol}^{\text{-1}}\text{)× (1.6×}\text{10}^{\text{-19}}\text{C)=}\text{ }\text{\textasciitilde115.6 C}$$

The identification and quantification of the products were determined from the calibration curves by applying standard solutions with known concentrations of commercially purchased pure reactants and final products. The Faradaic efficiency of BN (%), BN yield (mmol h^−1^), BA conversion (%), and BN selectivity (%) were calculated using equations (1-4), and F is the Faraday constant (96485 C mol^−1^):

$$\text{FE}\text{ }\left( \text{\%} \right)\text{ }\text{= }\text{ }\frac{\text{mol of formed}\text{ BN}}{\text{total passed }\text{charge}\text{ / (4×F)}}\text{ × }\text{100\% }\text{(1)}$$

$$\text{BN yield}\text{ }\left( \text{mmol }\text{h}^{\text{-1}} \right)\text{ }\text{=}\text{ }\frac{\text{mol of}\text{ }\text{formed}\text{ BN}}{\text{ }\text{BA }\text{oxidation reaction time}}\text{ }\text{(2)}$$

$$\text{BA conversion}\text{ }\left( \text{\%} \right)\text{ }\text{= }\text{ }\frac{\text{mol of}\text{ consumed}\text{ BA}\text{ }}{\text{mol of initial }\text{BA}}\text{ }\text{×}\text{ }\text{100\%}\text{ }\text{(3)}$$

$$\text{BN selectivity}\text{ }\left( \text{\%} \right)\text{ = }\frac{\text{mol of formed}\text{ BN}}{\text{mol of }\text{consumed}\text{ }\text{BA}}\text{ × 100\% (4)}$$

- 1. **In situ Raman measurements**

In situ Raman spectroscopy measurements were carried out using inVia-Reflex spectrometer (Renishaw) with a laser excitation wavelength of 532 nm, and the applied potential for the BAOR was in the range 0.2–0.5 V *vs.* Ag/AgCl controlled by an electrochemical workstation (CHI 660E). Self-supported NiMoO_4_/NF was used as working electrode, while Ag/AgCl electrode and Pt wire were applied as reference electrode and counter electrode, respectively. The electrolyte used was 1.0 M KOH with 10.0 mM BA. The Raman spectra at various applied potentials were collected after 120 s.

- 1. **In situ XAS measurements**

In situ Ni K-edge XANES spectra of NiMoO_4_ were collected at the BL14W1 station in Shanghai Synchrotron Radiation Facility (SSRF). The beam from the bending magnet was monochromatized utilizing a Si (111) double-crystal monochromator and further detuned by 30% to remove higher harmonics. The Ni K-edge XANES measurements of NiMoO_4_ were performed on carbon paper to avoid possible interference of the Ni signal from the nickel foam. Ag/AgCl electrode and Pt wire were used as reference electrode and counter electrode, respectively. The electrolyte used was 1.0 M KOH with 10.0 mM BA. The XANES spectra were collected using the fluorescence mode. To monitor the changes during the BAOR process, potentials in the range from OCP to 0.6 V *vs.* Ag/AgCl were applied for 10 min at each stage. During the measurements, the position of the absorption edge was calibrated using Ni foil and all the XAFS data were collected during one period of beam time.

- 1. **Computational Method**

Spin-polarized density functional theory (DFT) calculations were performed by the Vienna Ab-initio Simulation Package (VASP).^1,2^ The generalized gradient approximation (GGA) with a PerdewBurke-Ernzerhof (PBE) functional was used to describe the electronic exchange and correlation interaction.^3,4^ The plane-wave cutoff 400 eV was used and the van der Waals interactions were described by DFT-D3 method.^5^ In this work, the NiOOH surface structure was modeled using the *β*-phase of NiOOH derived from *β*–Ni(OH)_2_.^6^ The *β*-NiOOH (010) facet was constructed using 2×4×4 supercell containing 140 atoms. A vacuum region of 20 Å in thickness was set to avoid interactions between the periodic images. The Mo-NiOOH electrocatalyst was constructed *via* replacing one surface Ni atom with Mo atom. During the relaxation, the top two layers of atoms were relaxed, and the remaining atoms were fixed. The convergence thresholds of the electronic energy and the atomic force for the geometry optimizations were set to 5×10^−5^ eV and 0.05 eV/Å, respectively. A 1×1 ×1 Γ-centered Monkhorst–Pack mesh was adopted to sample the Brillouin zone integration. The Hubbard U correction of localized Ni was described with DFT+U method (U_eff_ = 5 eV) reported by Dudarev *et al.*^7^ The Poisson-Boltzmann implicit solvation model was used to describe the solvent effect, in which the dielectric constant was taken as 80 for water.^8^

1. **Supplementary Figures and Tables**

**
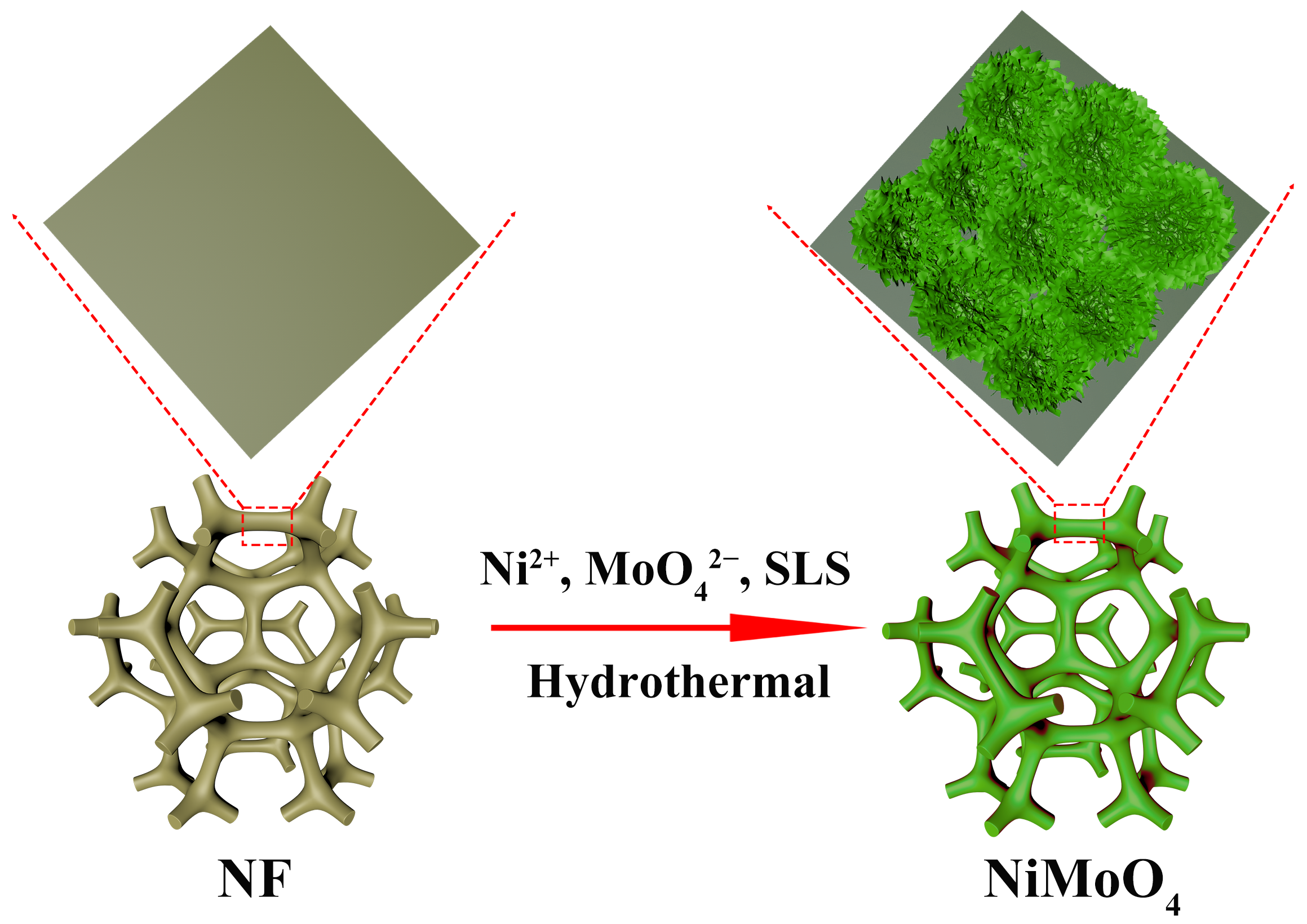
**

Figure S1. Schematic illustration of the synthesis process for NiMoO_4_ on Ni foam.


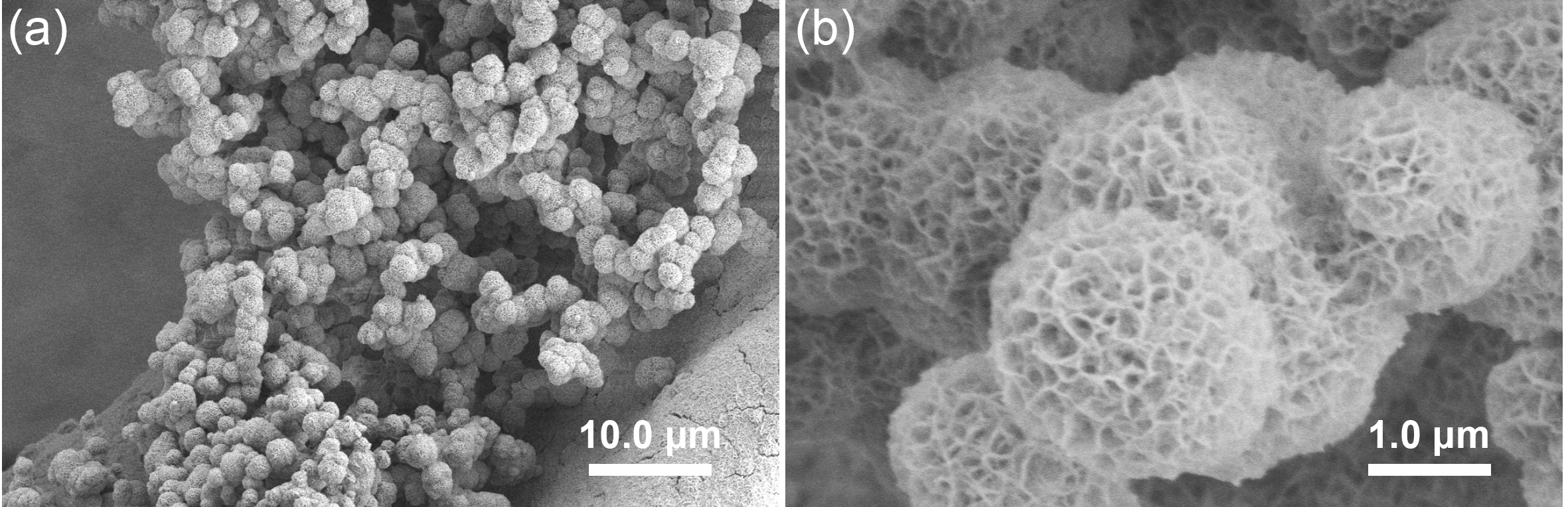


Figure S2. (a-b) SEM images of NiMoO_4_ with different resolutions.


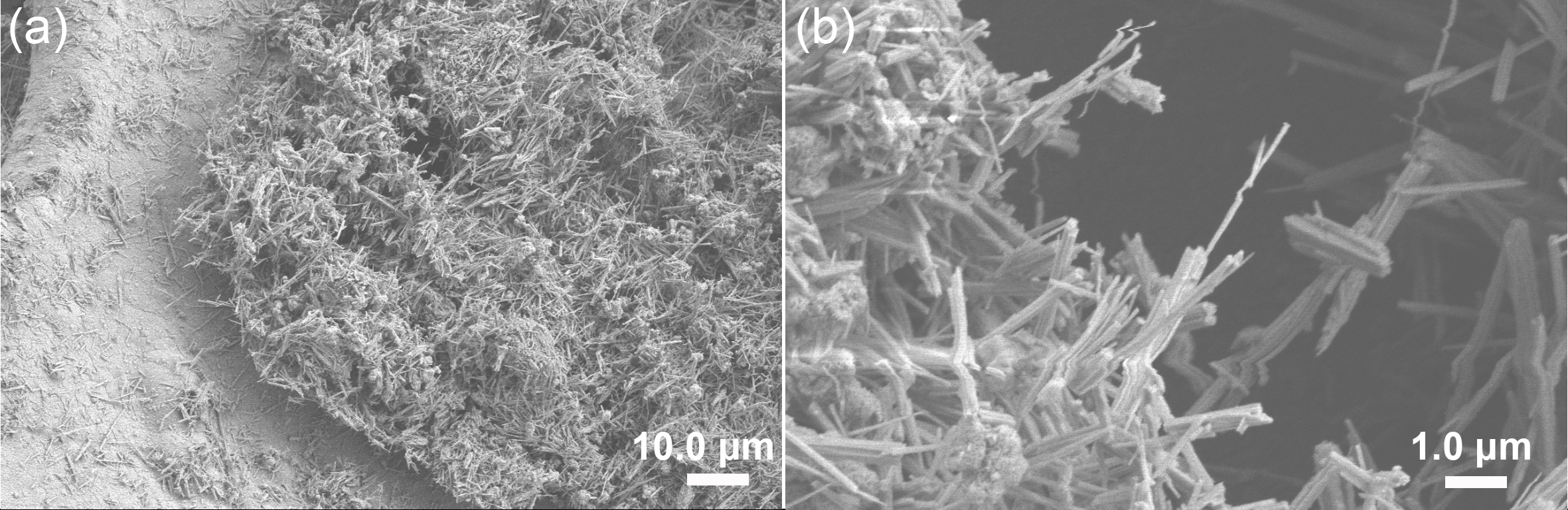


Figure S3. (a-b) SEM images of NiMoO_4_ sample prepared without the use of SLS.


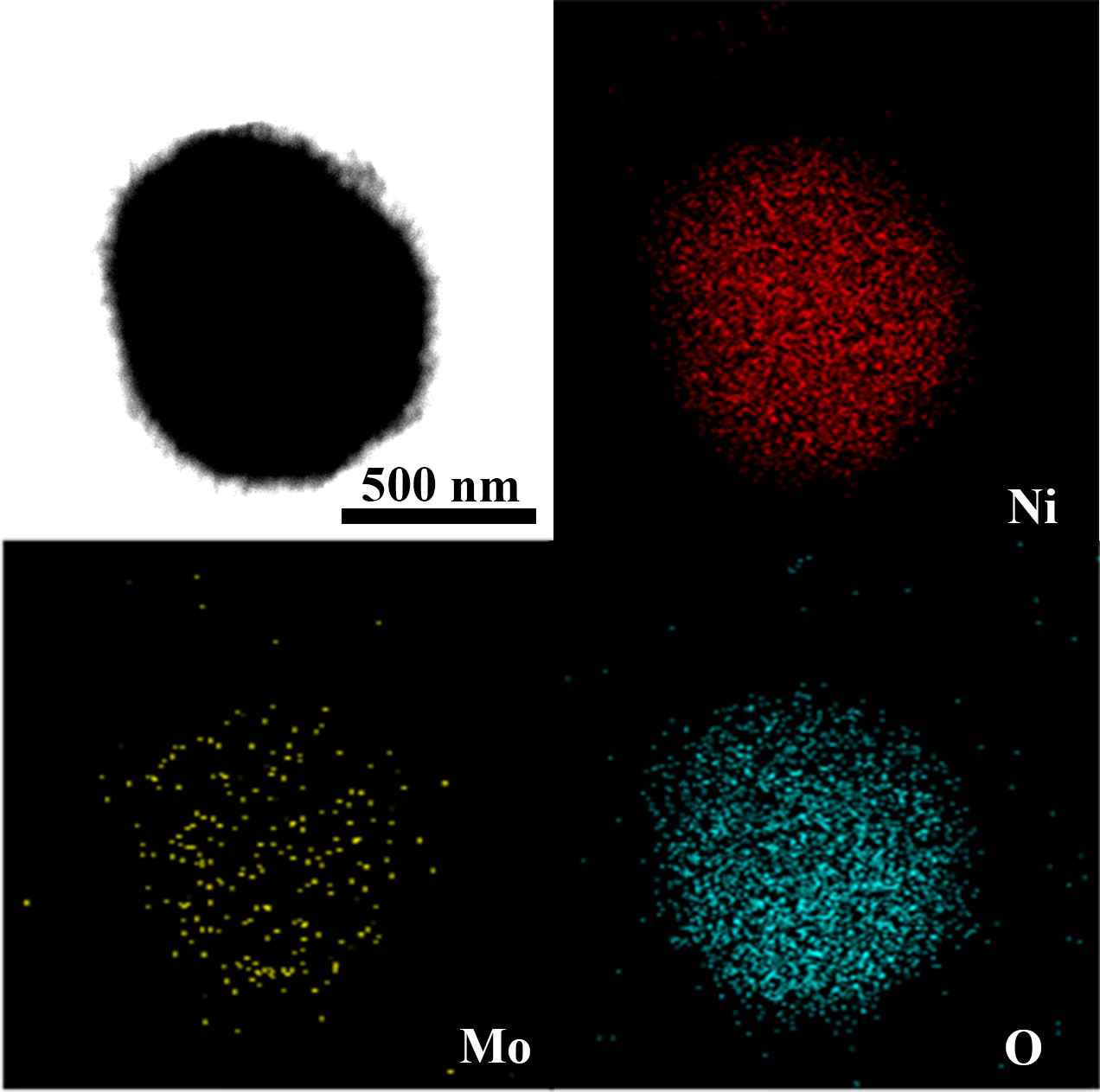


Figure S4. HAADF-STEM image and the corresponding elemental mapping of NiMoO_4_.

**
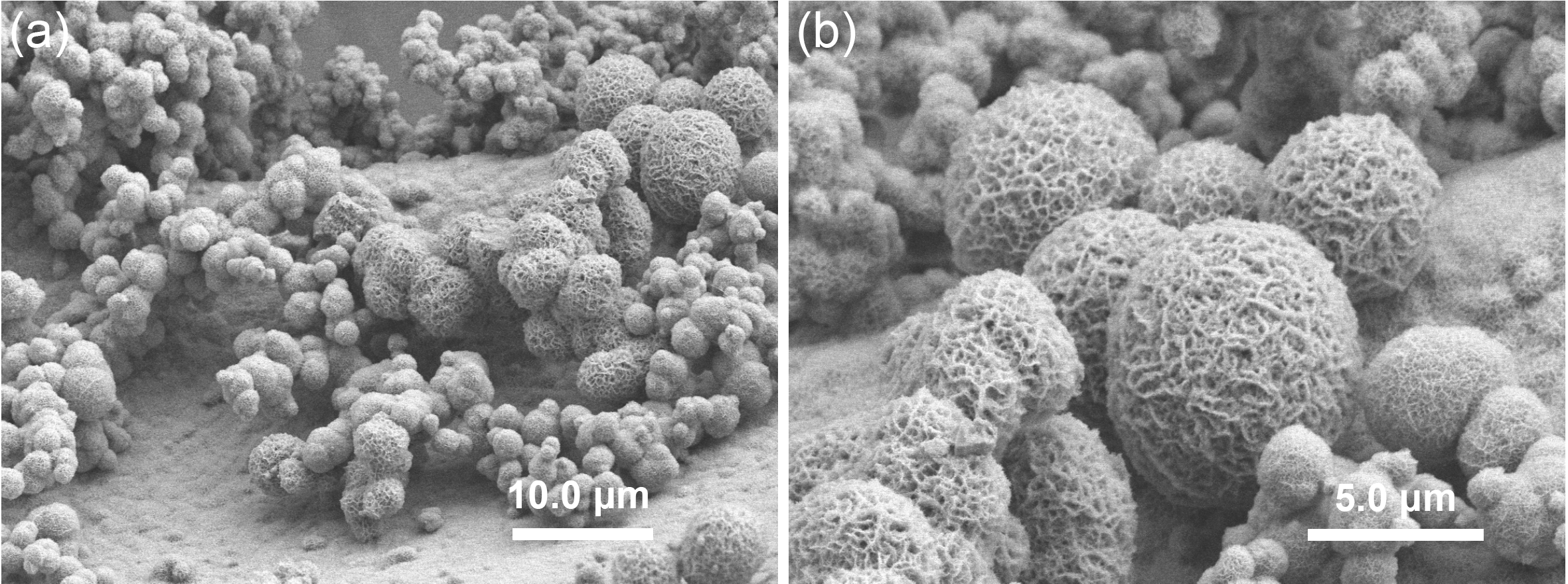
**

Figure S5. (a-b) SEM images of Ni(OH)_2_ with different resolutions.

**
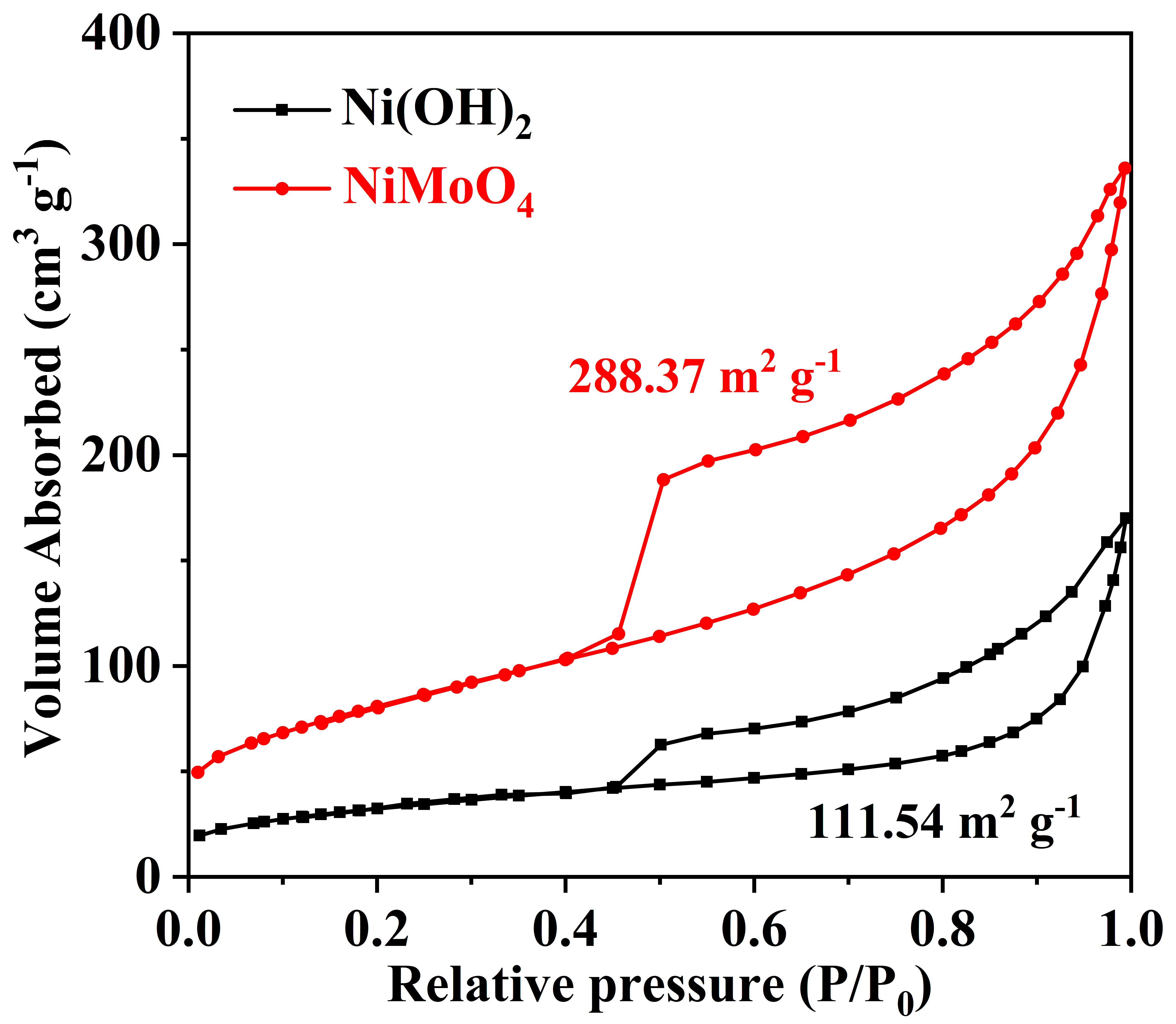
**

Figure S6. N_2_ adsorption–desorption isotherms of NiMoO_4_ and Ni(OH)_2_.

**
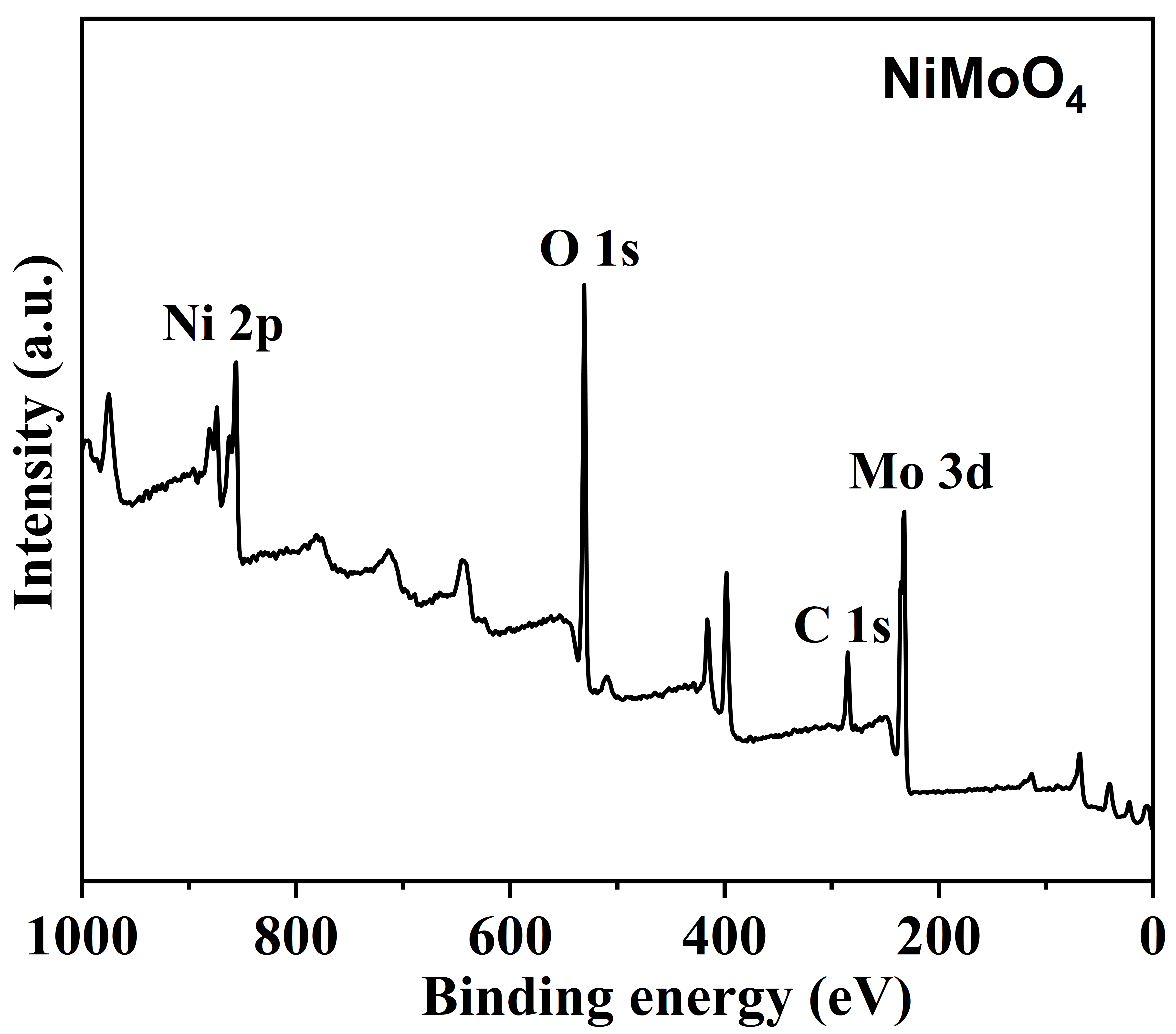
**

Figure S7. XPS survey spectrum of NiMoO_4_.


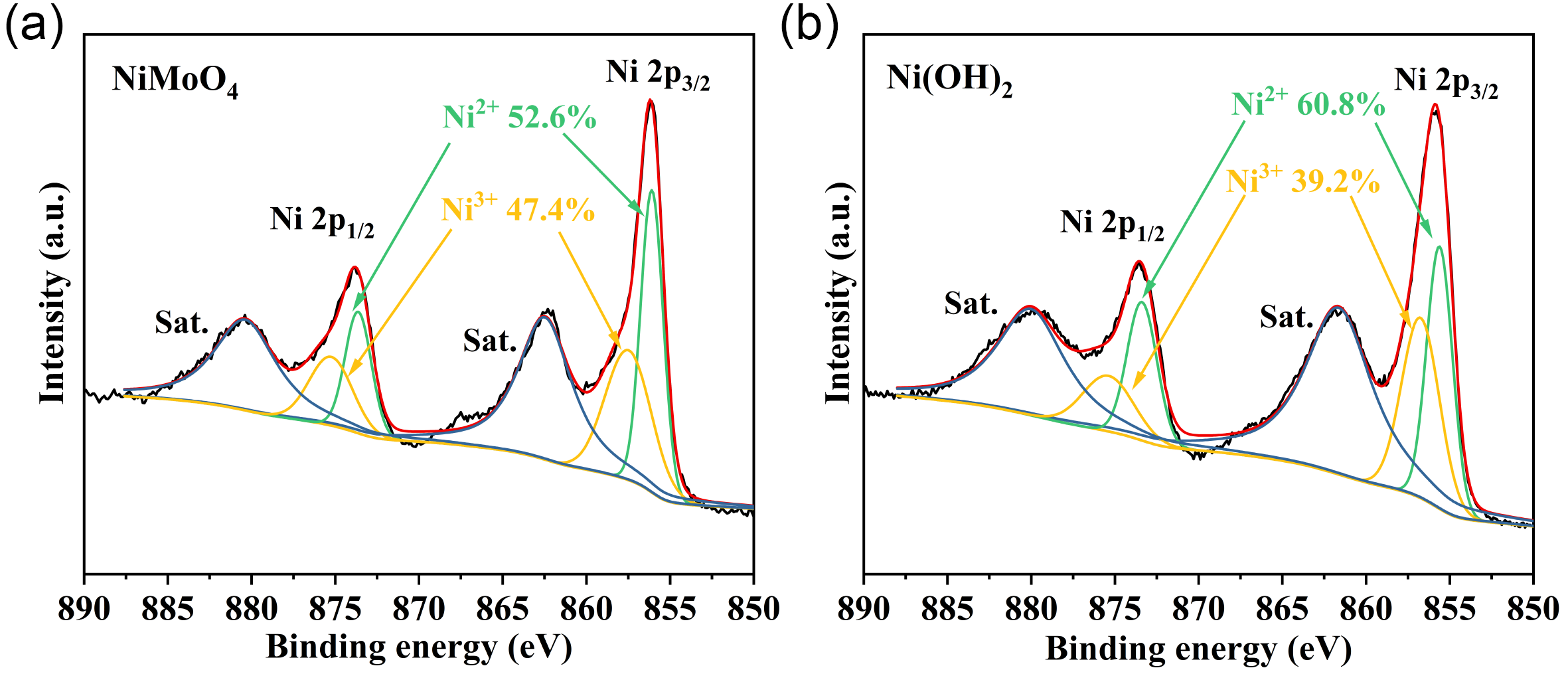


Figure S8. XPS Ni 2p spectra of (a) NiMoO_4_, and (b) Ni(OH)_2_.


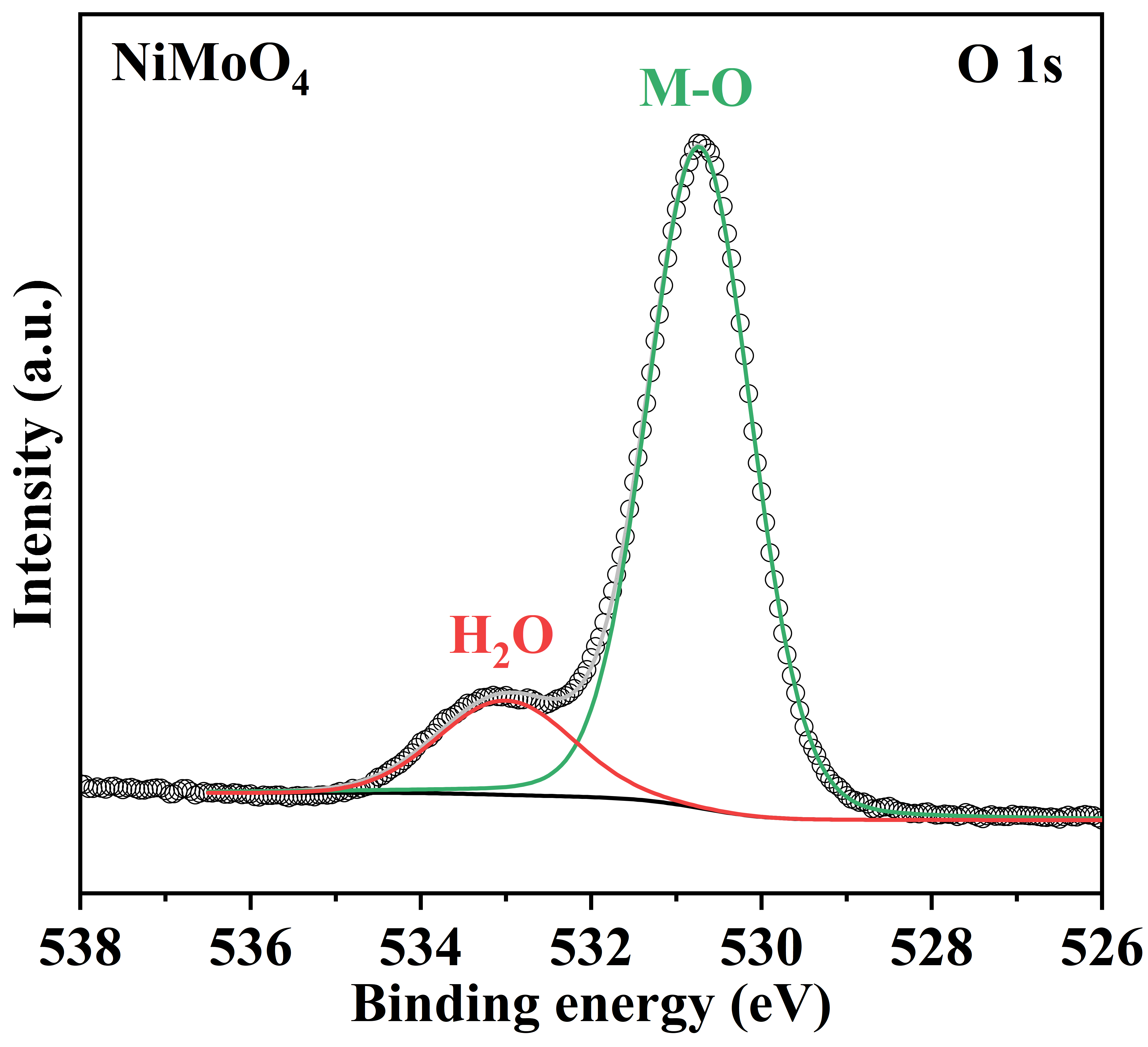


Figure S9. XPS O 1s spectrum of NiMoO_4_.


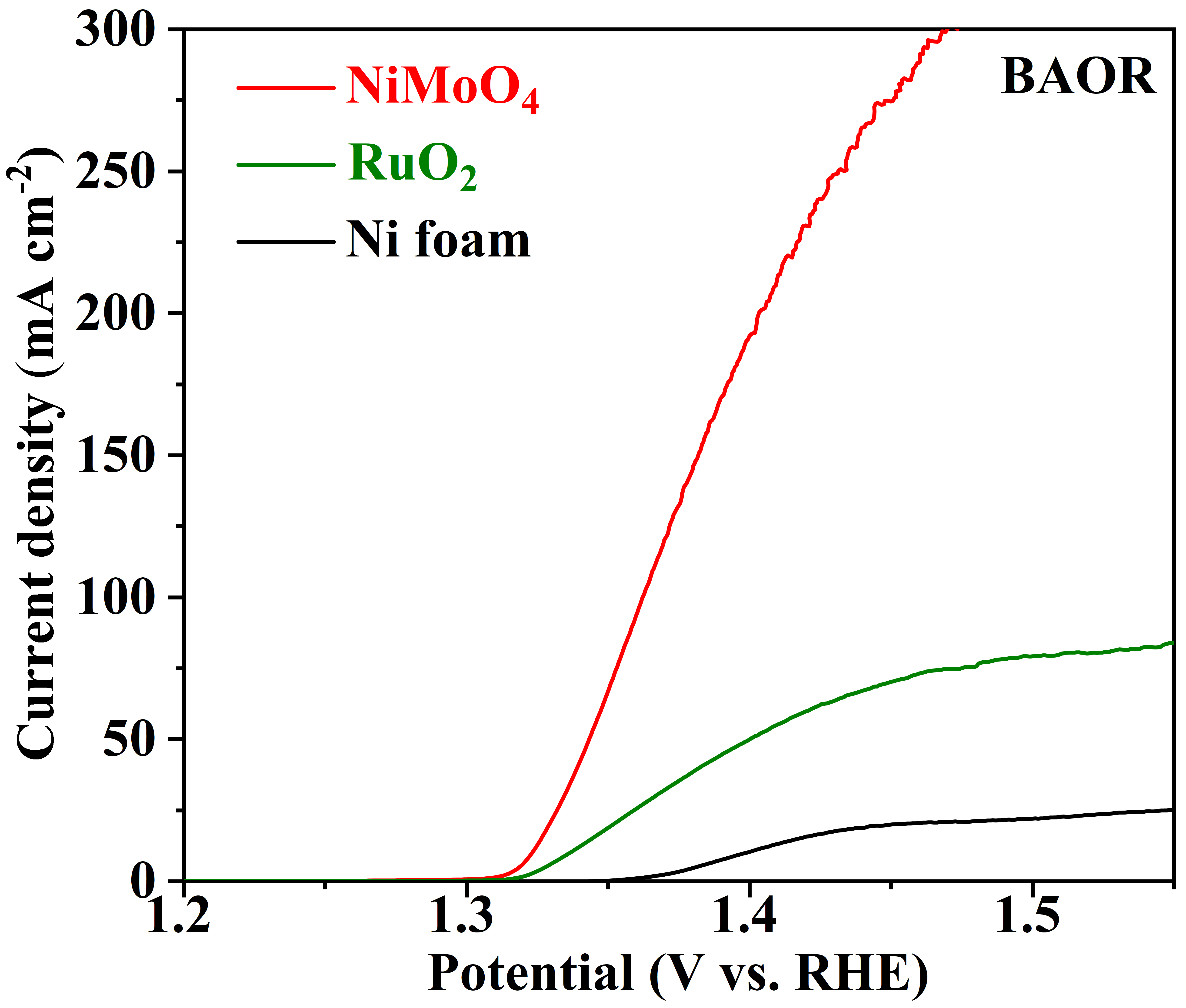


Figure S10. LSV curves of NiMoO_4_, commercial RuO_2_, and bare Ni foam in 1.0 M KOH with 10.0 mM BA.

**
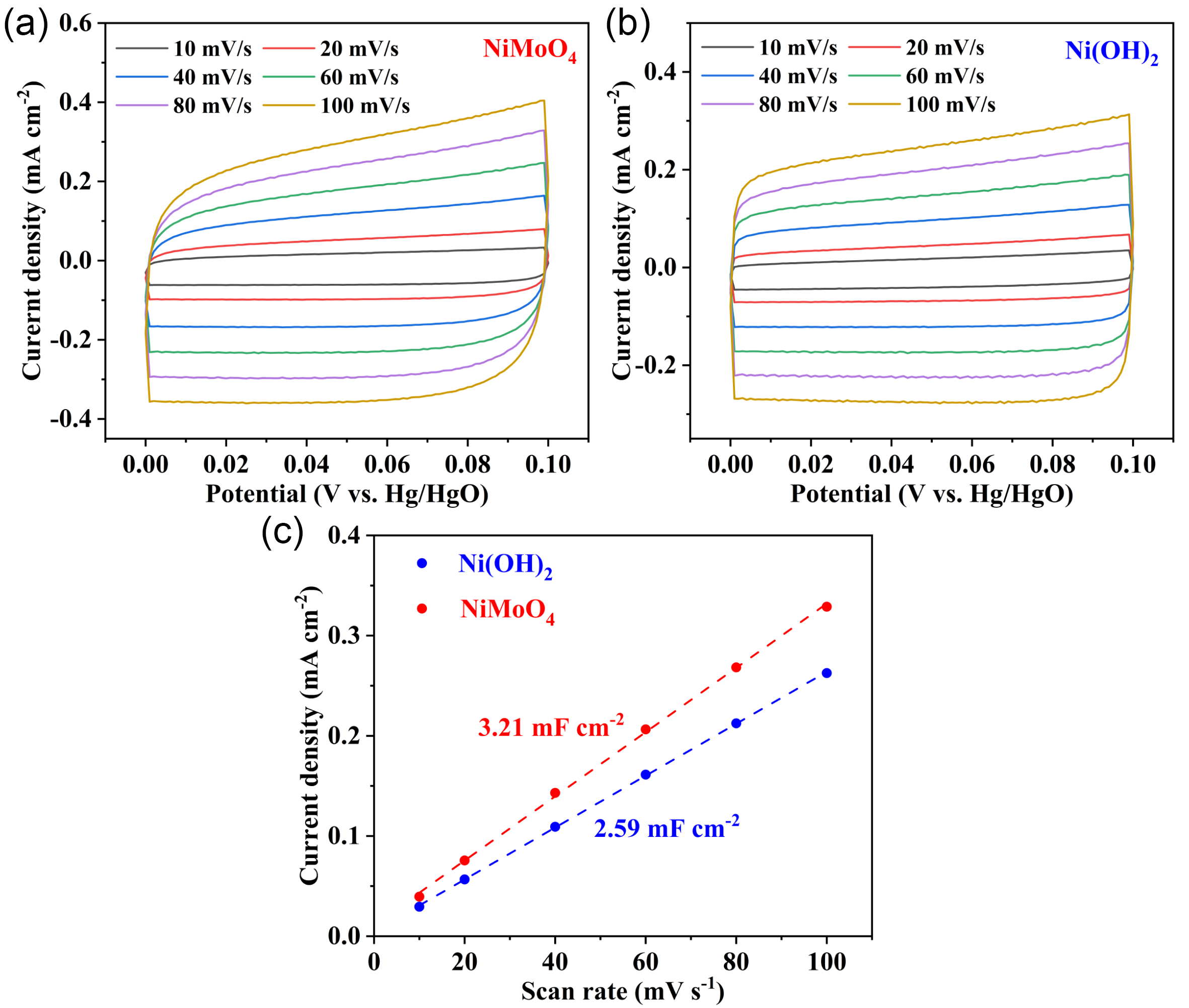
**

Figure S11. (a) CV curves for NiMoO_4_ sample at the scan rates from 10 to 100 mV/s, (b) CV curves for Ni(OH)_2_ sample at the scan rates from 10 to 100 mV/s, (c) Current density as a function of the scan rate to give the double-layer capacitance (C_dl_) for NiMoO_4_ and Ni(OH)_2_.

**
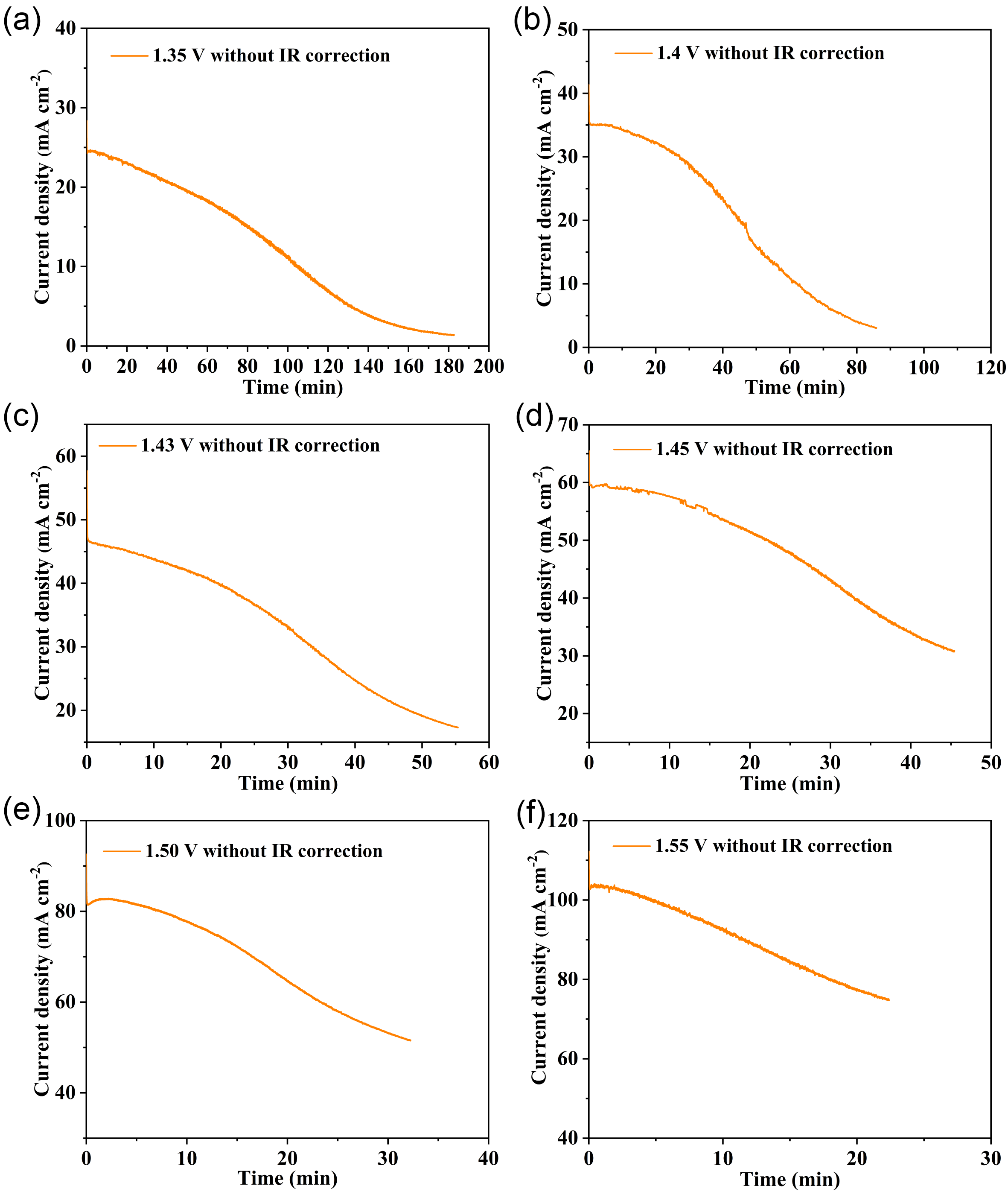
**

Figure S12. (a-f) Chronoamperometry curves of NiMoO_4_ with the same total passing charge of 115.6 C at different potentials (*vs.* RHE) in 1.0 M KOH containing 10.0 mM BA.

**
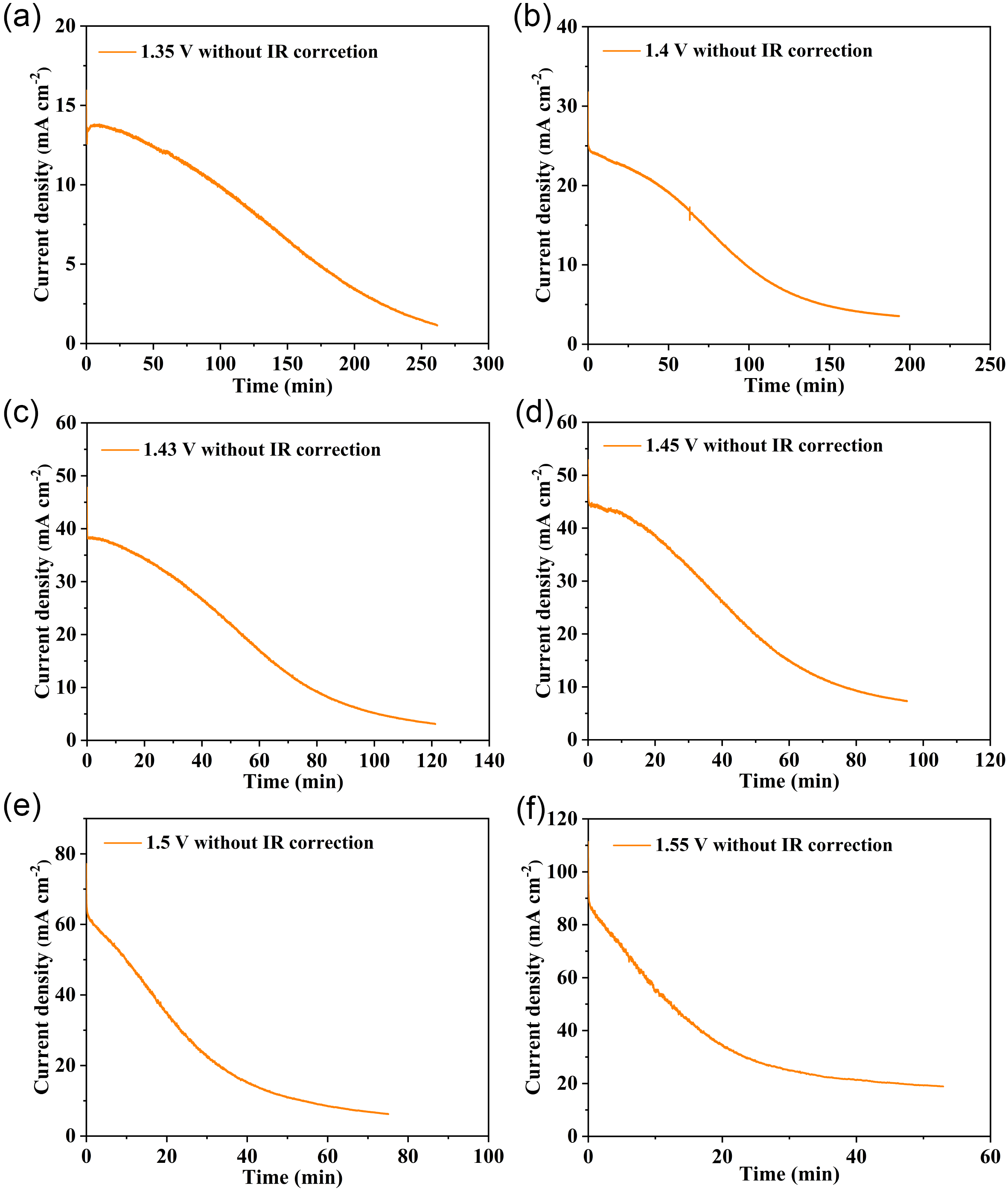
**

Figure S13. (a-f) Chronoamperometry curves of Ni(OH)_2_ with the same total passing charge of 115.6 C at different potentials (*vs.* RHE) in 1.0 M KOH containing 10.0 mM BA.

**
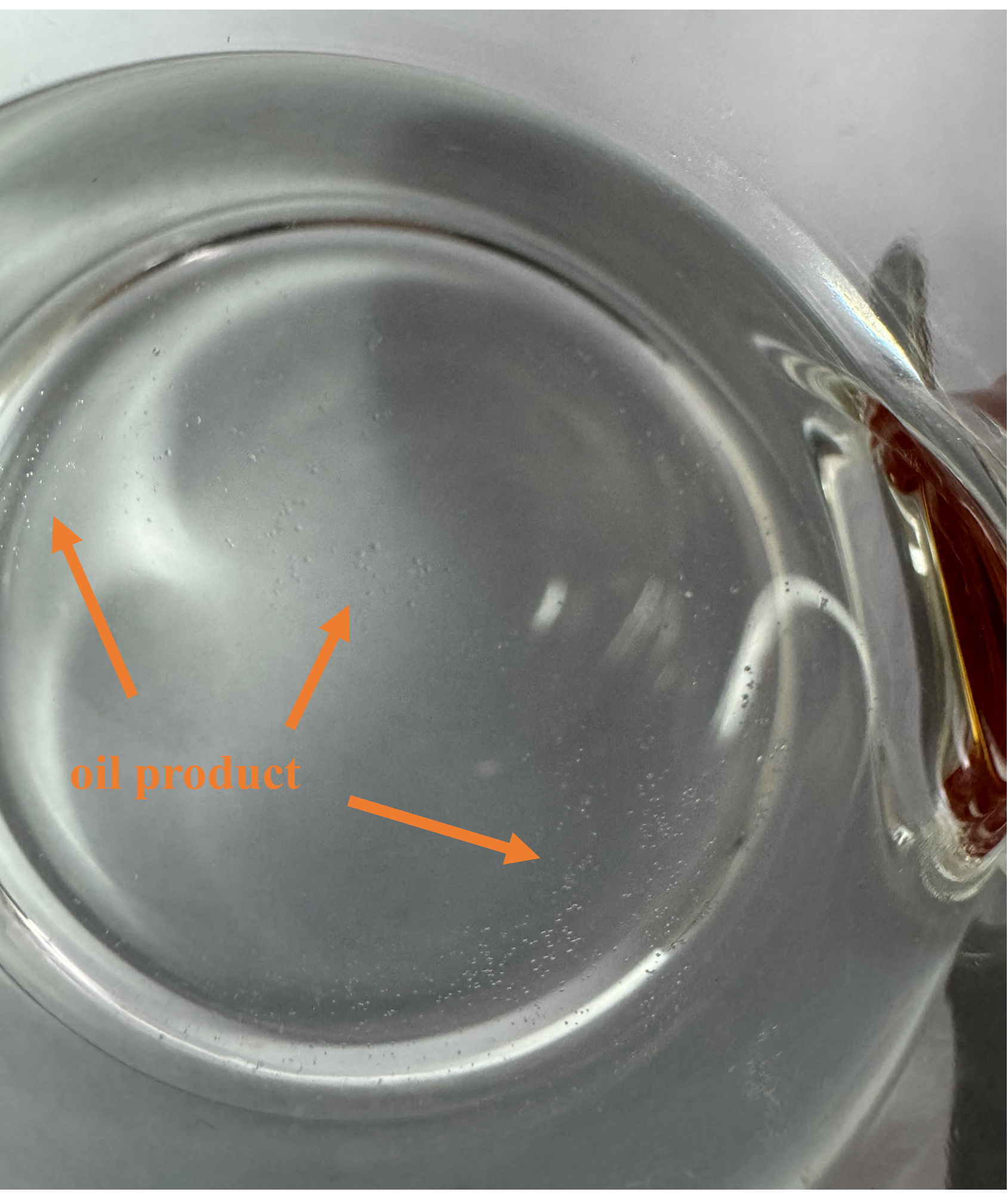
**

Figure S14. The optical photograph of the oil product floated on the electrolyte surface after BAOR.

**
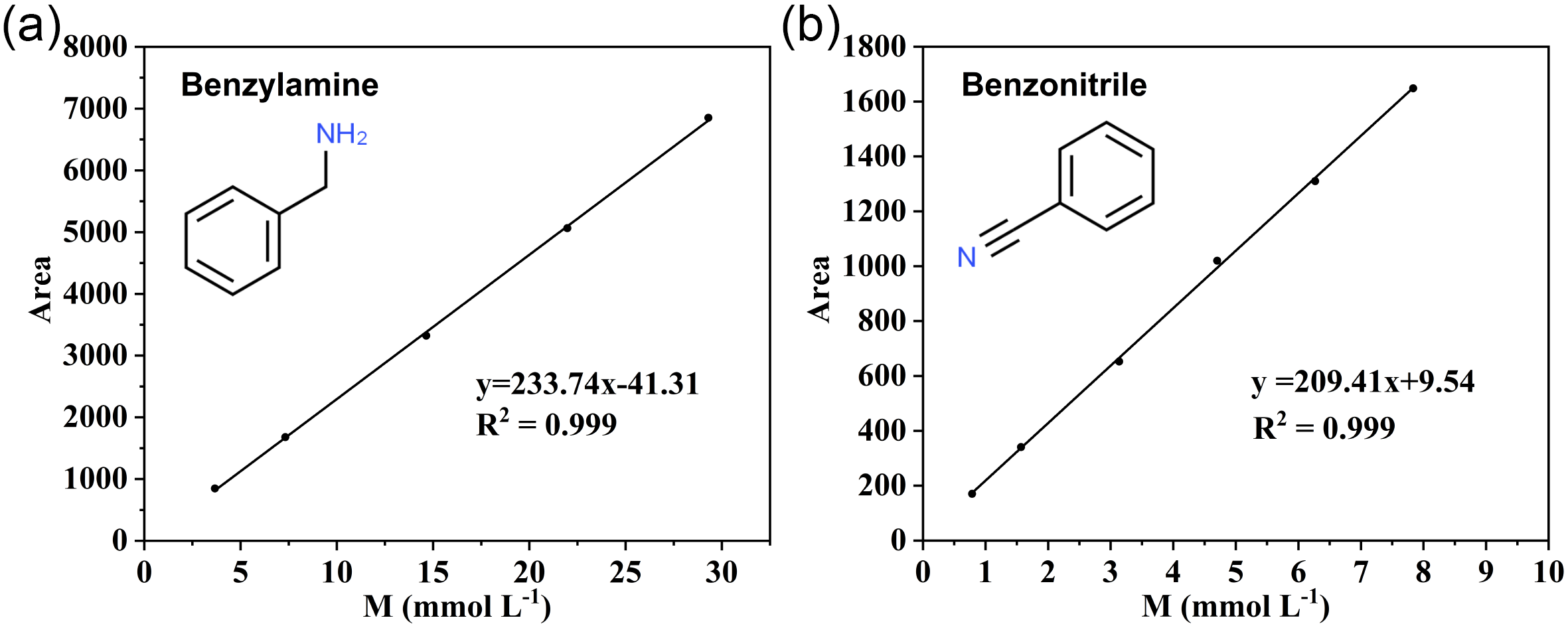
**

Figure S15. GC calibration curves for (a) benzylamine (BA), and (b) benzonitrile (BN).

**
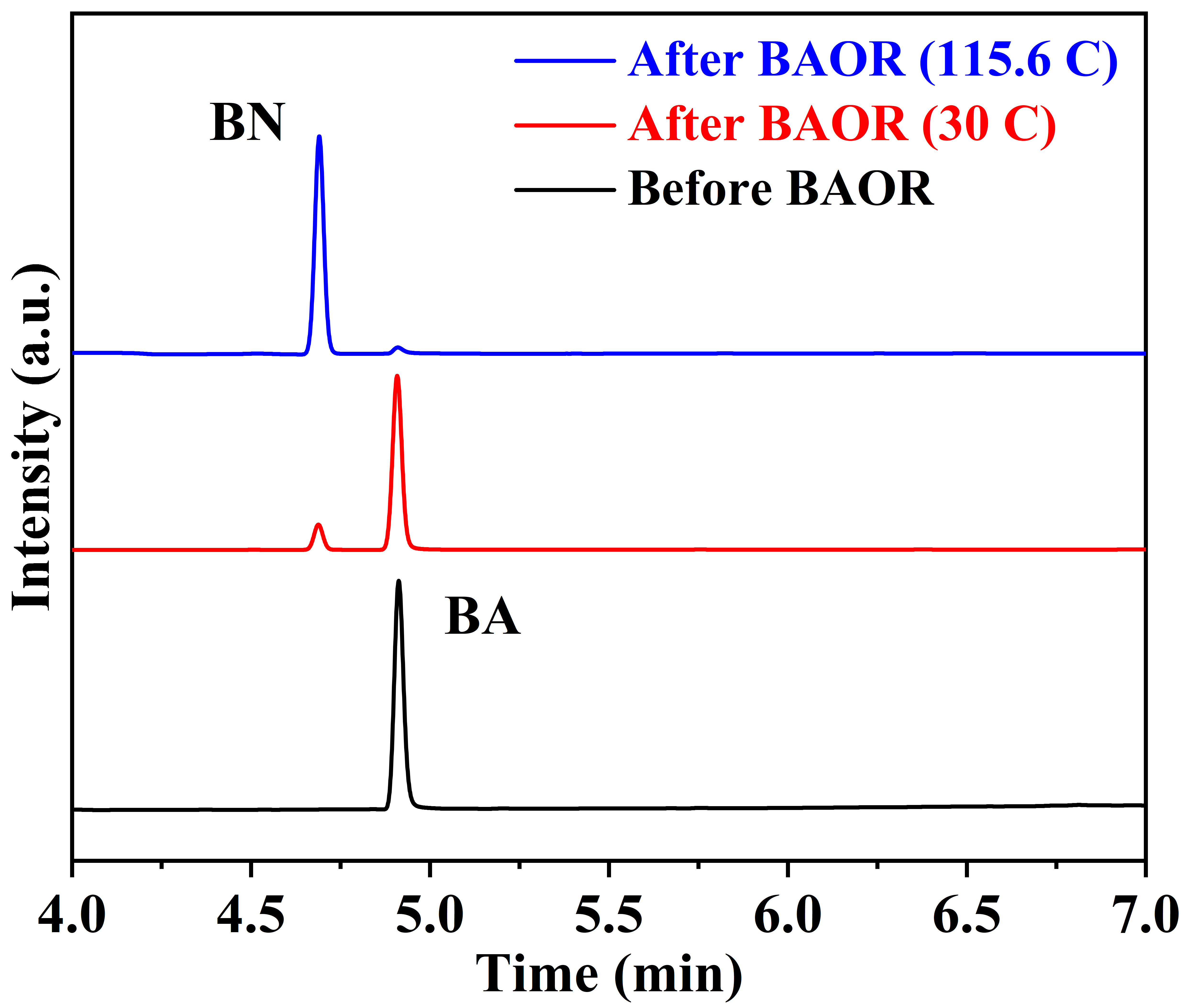
**

Figure S16. GC signals obtained at different reaction stages.


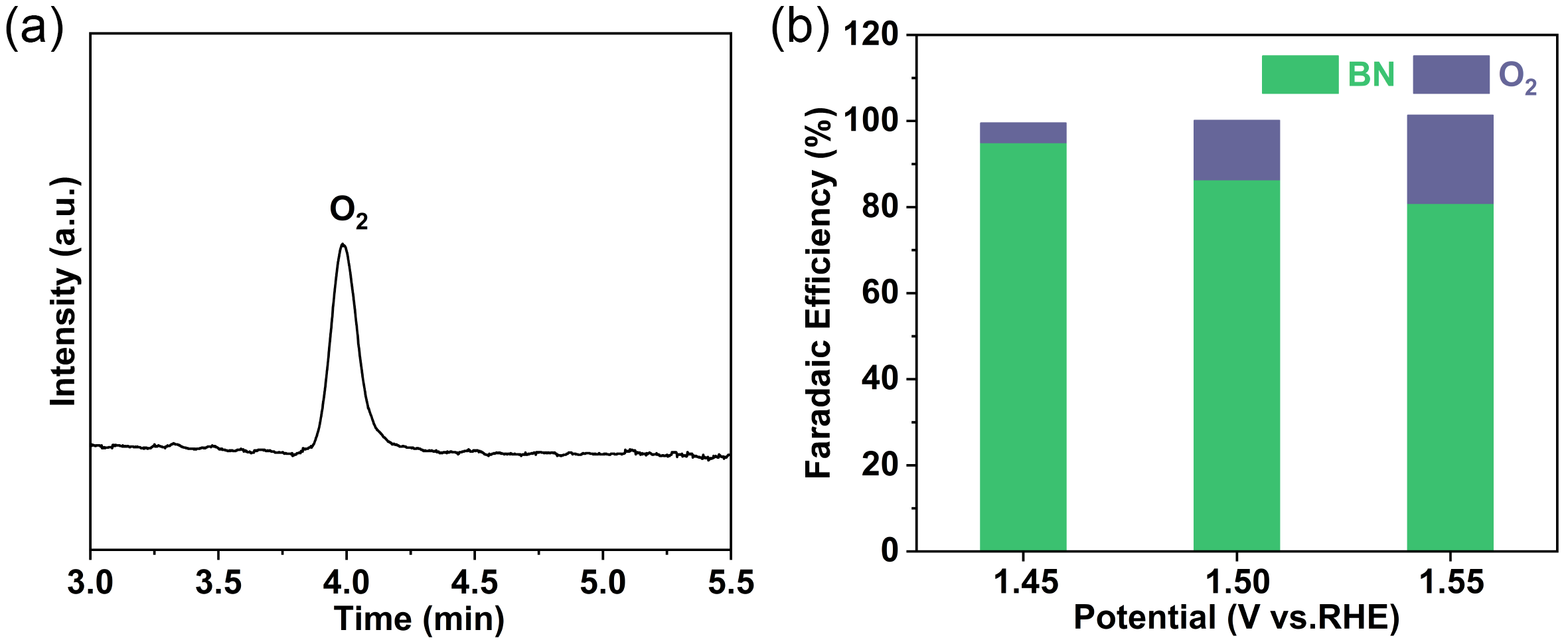


Figure S17. (a) The typical GC plot for O_2_ generated at the anode of two-chambered H-type electrolytic cell, (b) FEs of BN, and O_2_ on NiMoO_4_ electrode at various potentials.

**
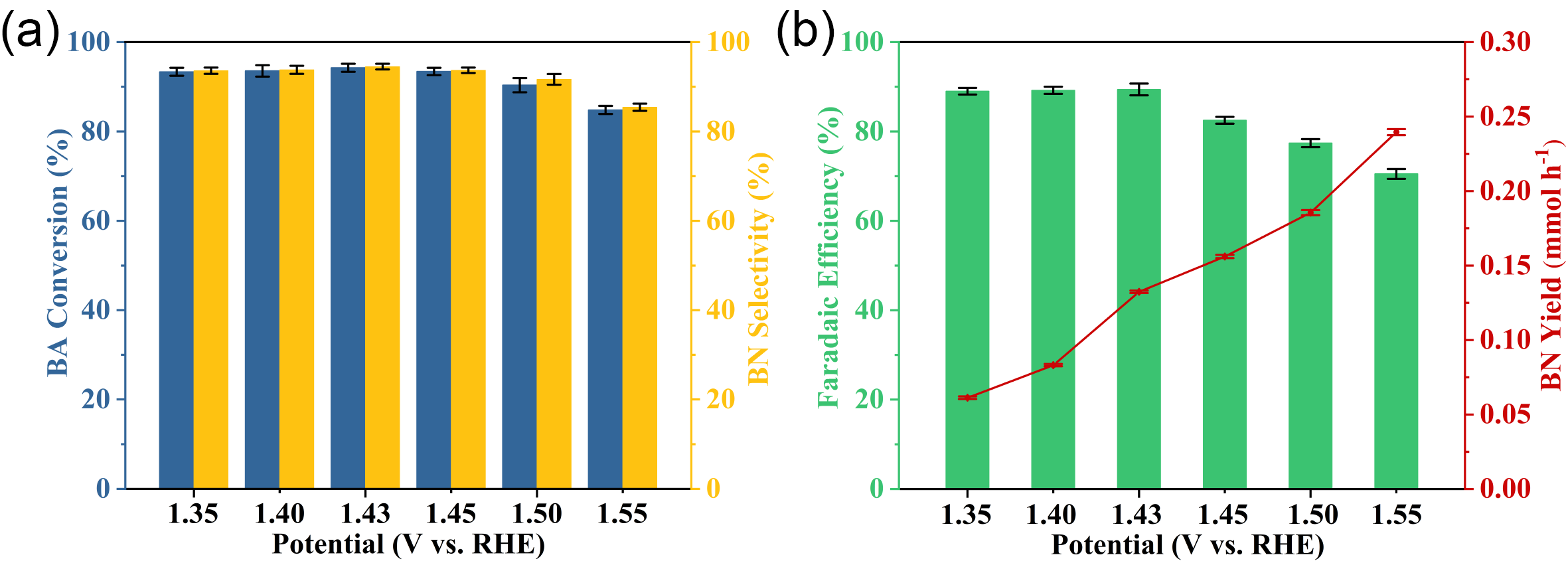
**

Figure S18.(a) BA conversion, and BN selectivity of Ni(OH)_2_ at various potentials. (b) FE of BN, and BN yield of Ni(OH)_2_ at various potentials.

**
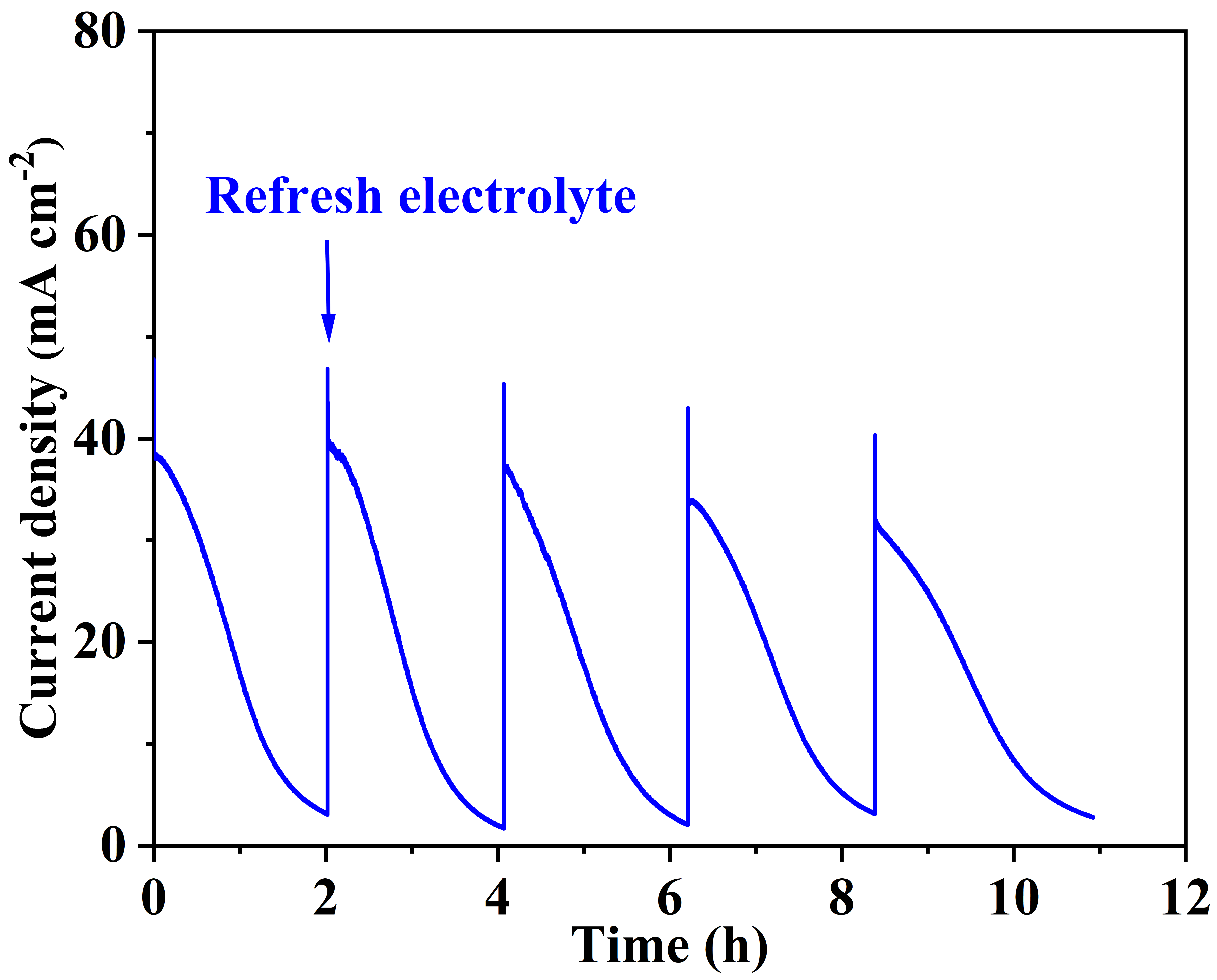
**

Figure S19. The successive chronoamperometry test of Ni(OH)_2_ at 1.43 V *vs.* RHE for 5 cycles.

**
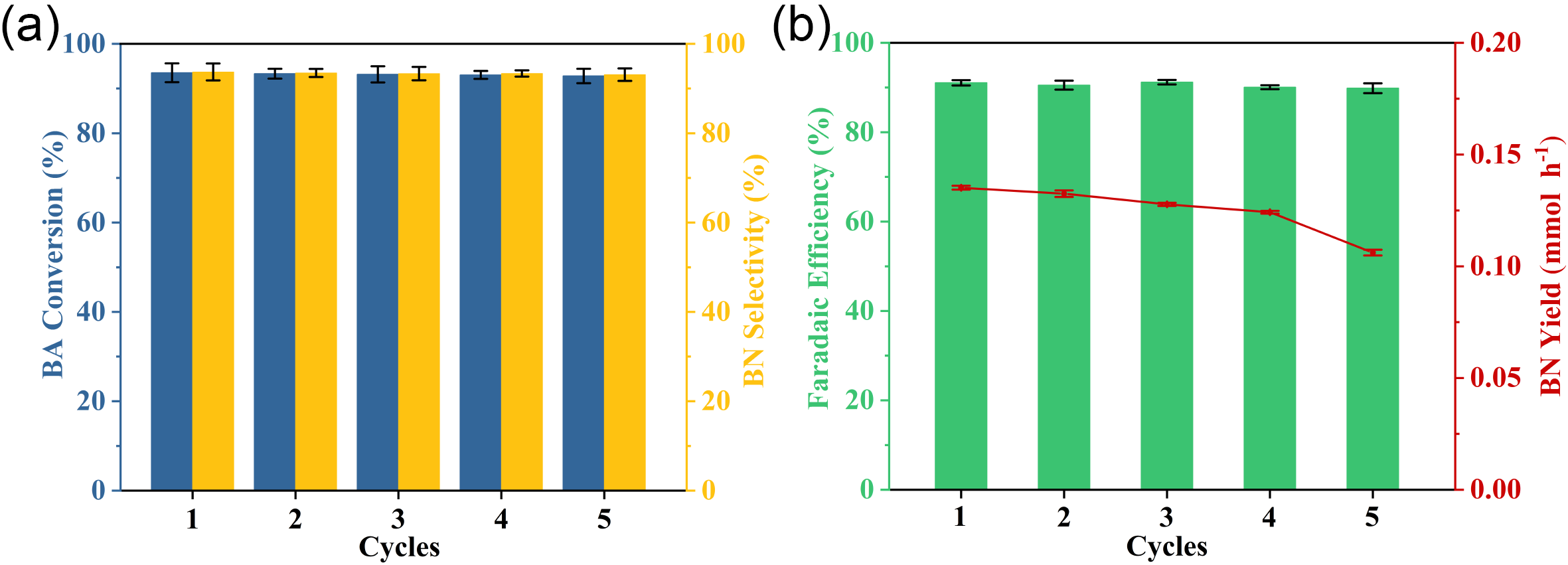
**

Figure S20. (a) BA conversion, and BN selectivity of Ni(OH)_2_ for 5 cycles. (b) FE of BN, and BN yield of Ni(OH)_2_ for 5 cycles.


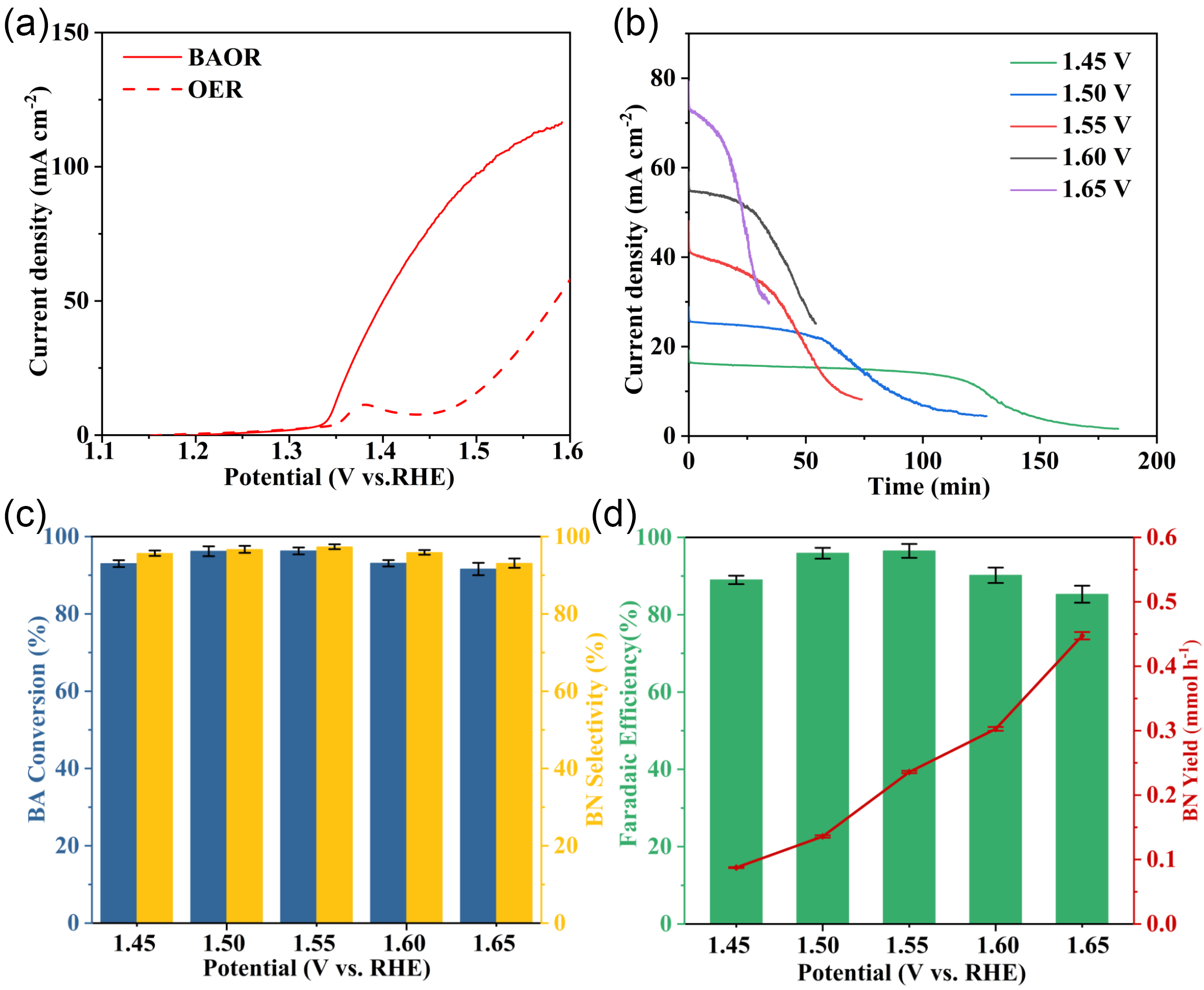


Figure S21. (a) LSV curves of NiMoO_4_ in 0.1 M KOH/0.5 M Na_2_SO_4_ with or without 10.0 mM BA. (b) Chronoamperometry curves of NiMoO_4_ with the same total passing charge of 115.6 C at different potentials (*vs.* RHE) without IR correction in 0.1 M KOH/0.5 M Na_2_SO_4_ with 10.0 mM BA. (c) BA conversion, and BN selectivity of NiMoO_4_ at various potentials. (d) FE of BN, and BN yield of NiMoO_4_ at various potentials.


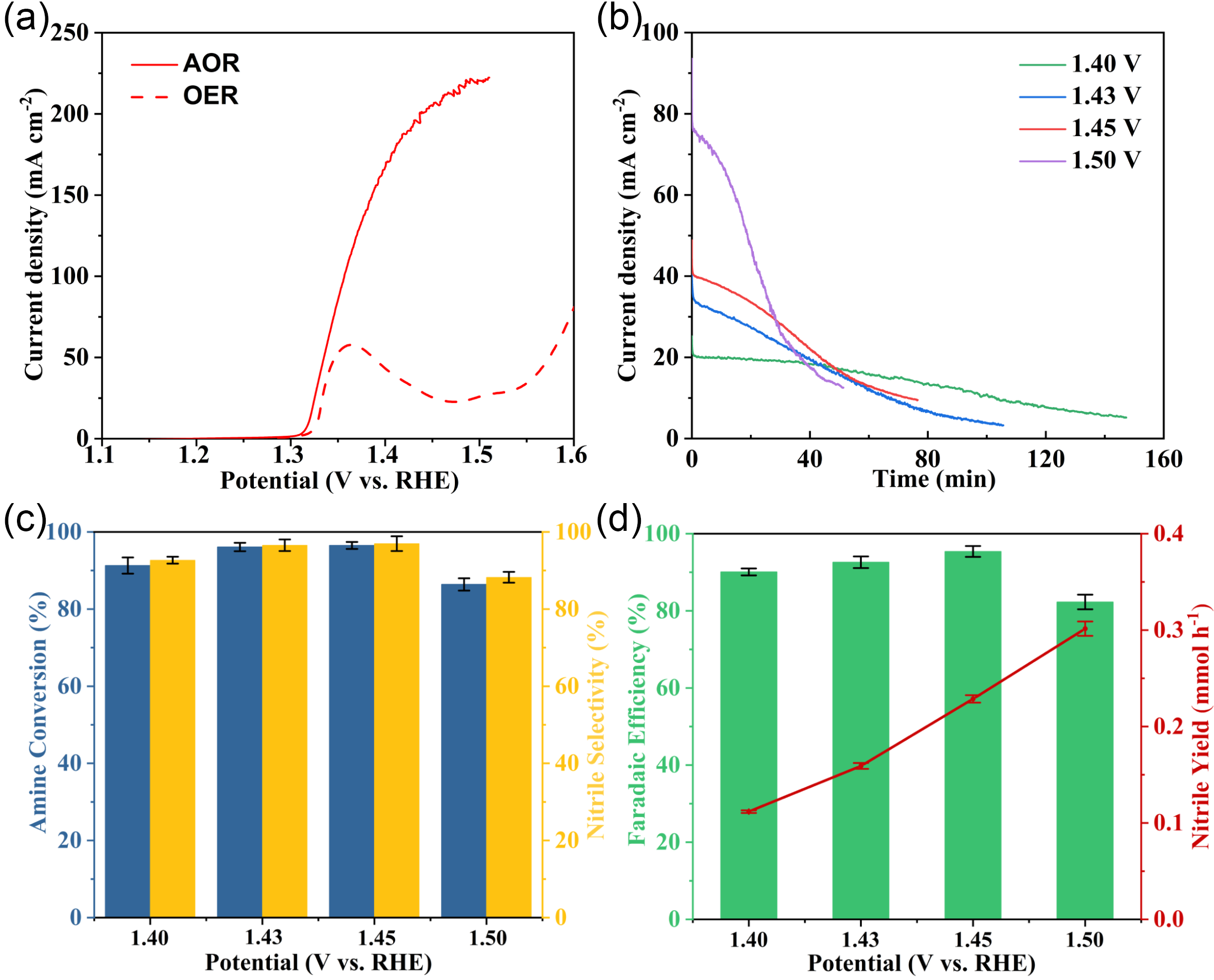


Figure S22. (a) LSV curves of NiMoO_4_ in 1.0 M KOH with or without 10.0 mM cyclohexenylethylamine. (b) Chronoamperometry curves of NiMoO_4_ with the same total passing charge of 115.6 C at different potentials (*vs.* RHE) without IR correction in 1.0 M KOH with 10.0 mM cyclohexenylethylamine. (c) Amine (cyclohexenylethylamine) conversion, and nitrile (cyclohexenylacetonitrile) selectivity of NiMoO_4_ at various potentials. (d) FE of nitrile, and nitrile yield of NiMoO_4_ at various potentials.


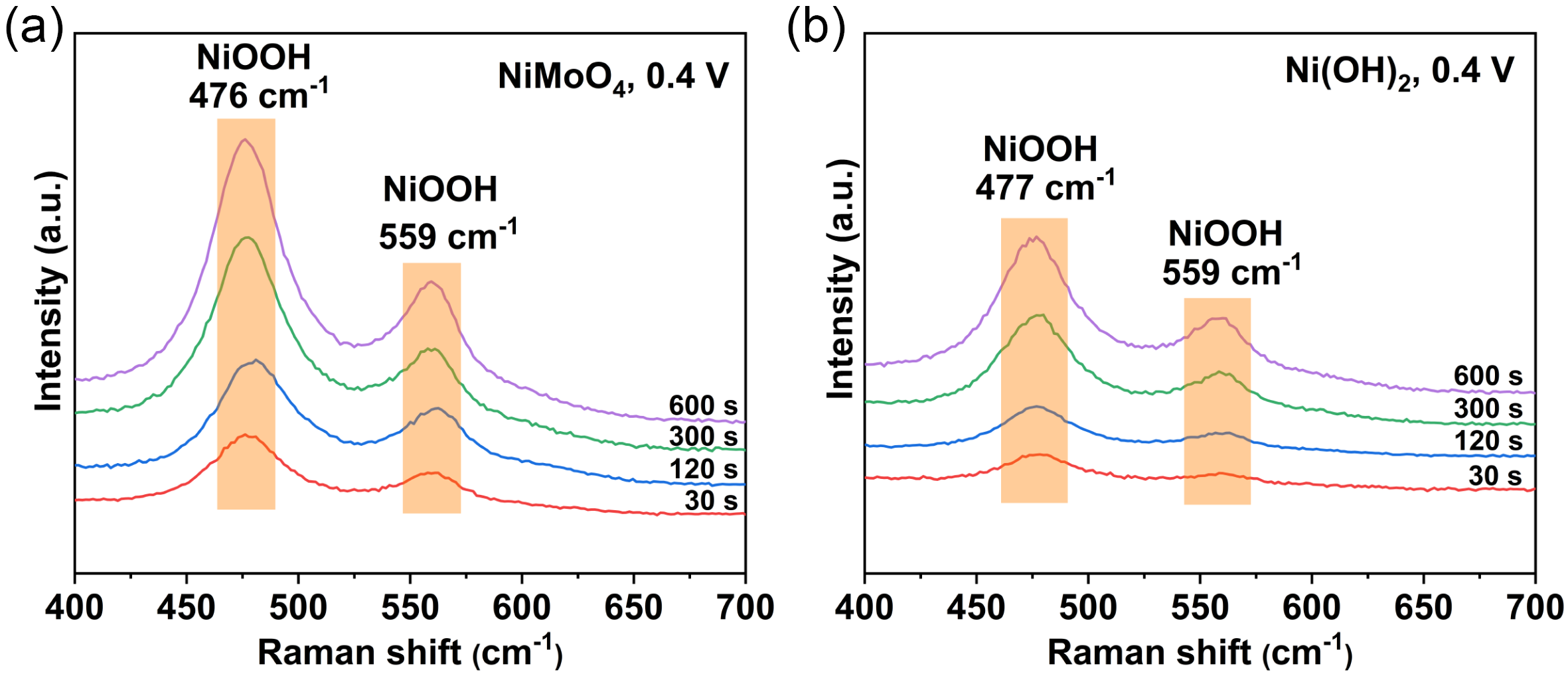


Figure S23. (a) In situ Raman spectra of NiMoO_4_ electrode collected at 0.4 V *vs.* Ag/AgCl with different reaction times in 1.0 M KOH with 10.0 mM BA, (b) In situ Raman spectra of Ni(OH)_2_ electrode collected at 0.4 V *vs.* Ag/AgCl with different reaction times in 1.0 M KOH with 10.0 mM BA.

**
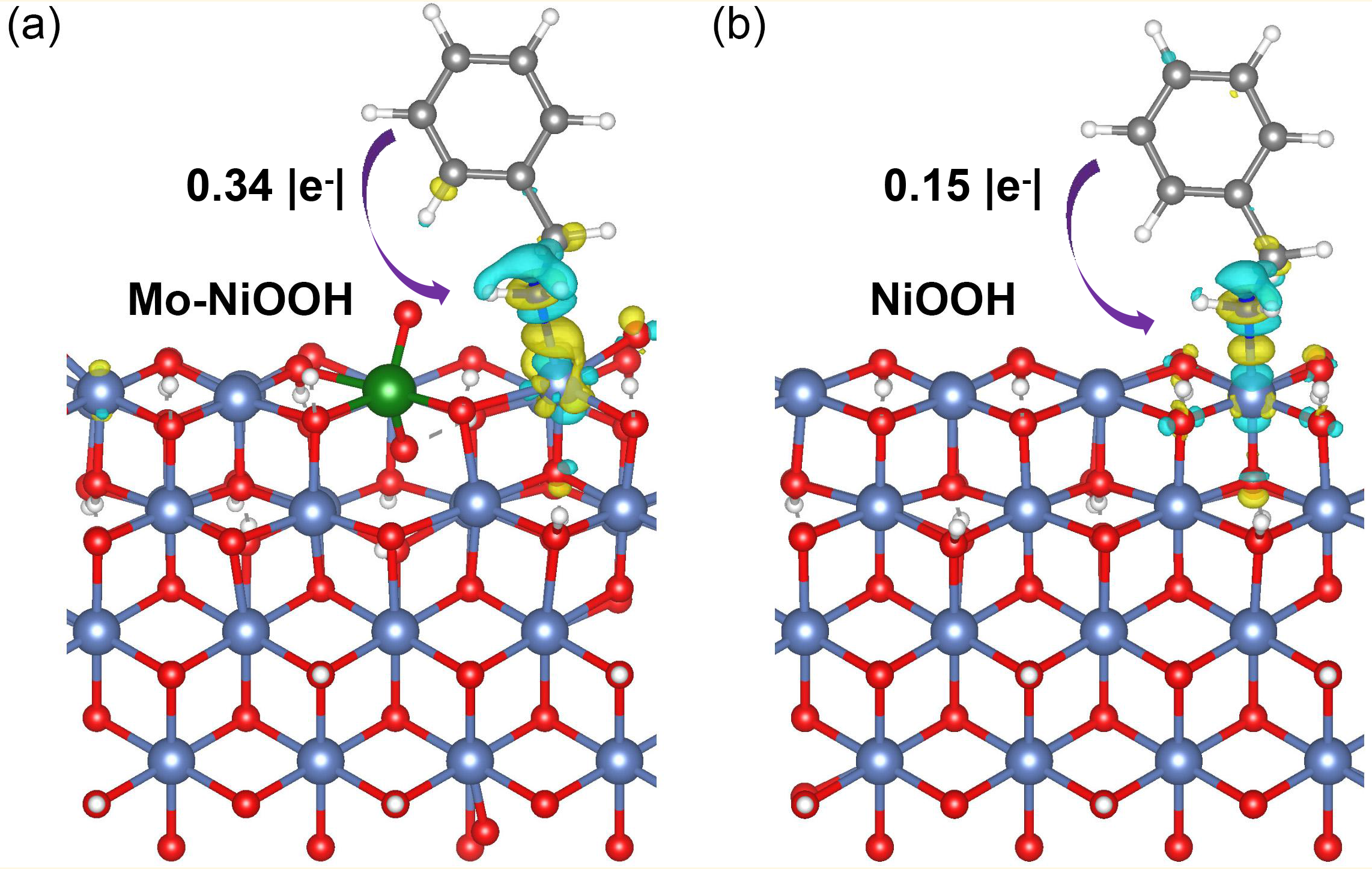
**

Figure S24. The charge density difference for (a) Mo-NiOOH, and (b) NiOOH. The isosurface level is set to be 0.005 e Å^−3^. Yellow means electron accumulation, and blue represent electron depletion.


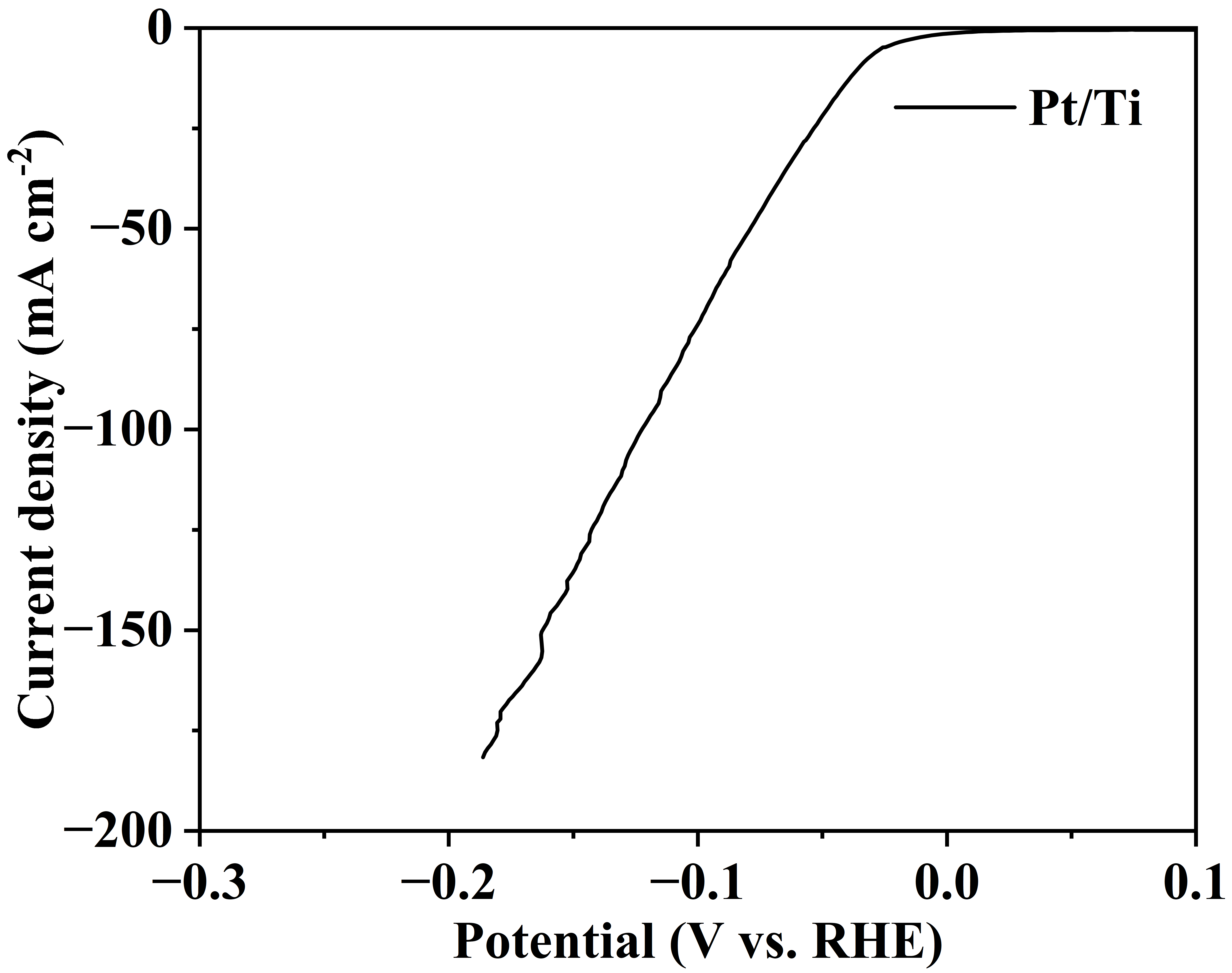


Figure S25. LSV curve of cathodic Pt/Ti fiber felt in 1.0 M KOH.


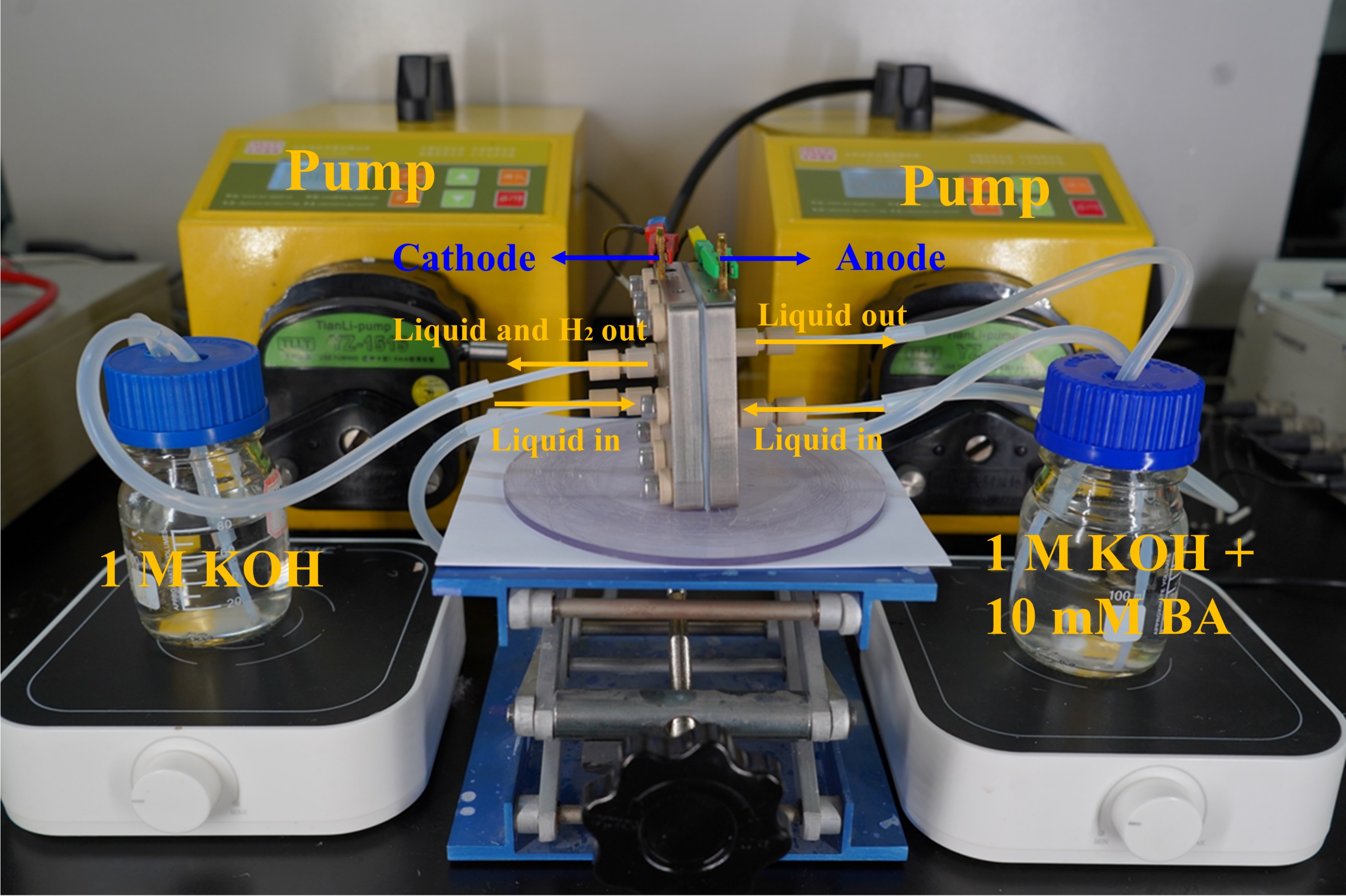


Figure S26. The optical photograph of the continuous-flow MEA reactor in the lab.

**
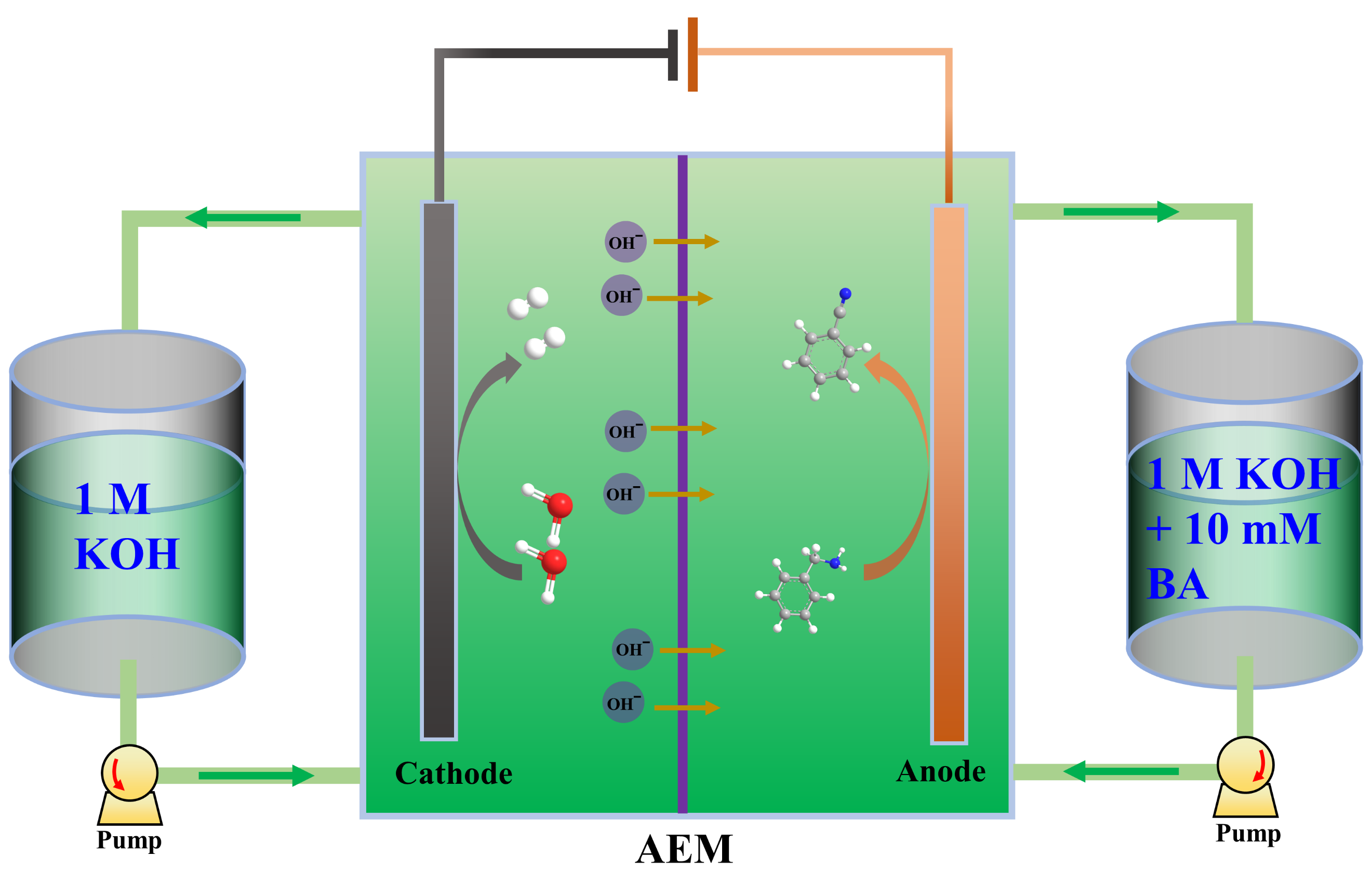
**

Figure S27. Schematic illustration of the two-electrode system in the continuous-flow reactor. The anode is for BA oxidation reaction (BAOR), while the cathode is for hydrogen evolution reaction (HER).


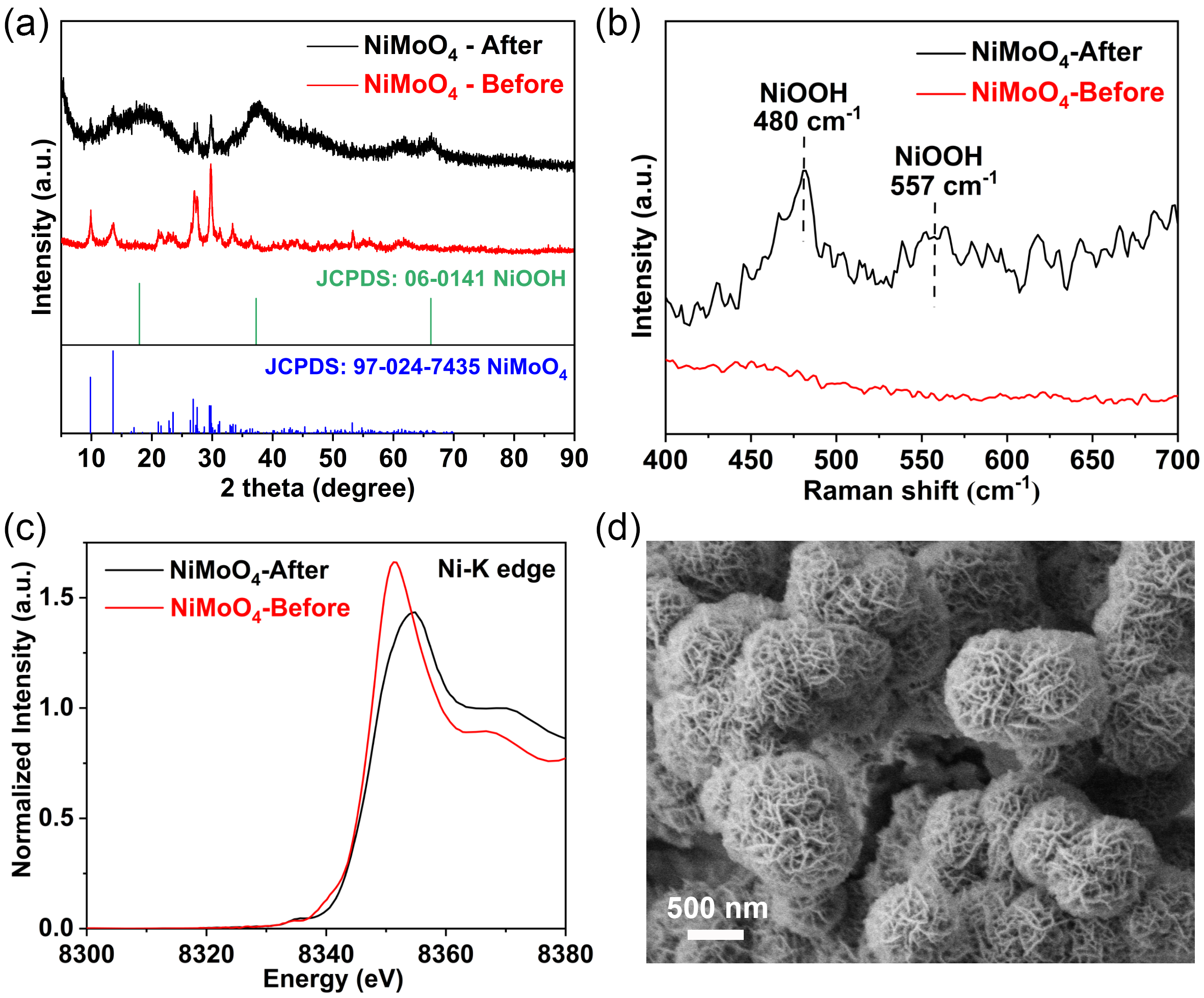


Figure S28. (a) XRD patterns of NiMoO_4_ powder before and after BAOR cycling test. (b) Raman spectra of NiMoO_4_ on nickel foam before and after BAOR cycling test. (c) Ni K-edge XANES spectra of NiMoO_4_ on nickel foam before and after BAOR cycling test. (d) SEM image of NiMoO_4_ on nickel foam after BAOR cycling test.

Table S1. Comparison of BAOR performance of NiMoO_4_ with some representative catalysts for amine electrooxidation in alkaline solutions.

| **Catalyst** | **Substrate** | **Applied potential (V *vs.* RHE)** | **Current density**  **( mA cm^−2^)** | **Amine conversion** | **Faradaic efficiency of** **nitrile** | **Ref.** |
| --- | --- | --- | --- | --- | --- | --- |
| NiMoO_4_ | benzylamine | 1.4 | 192.6 | 96.5% | 98.6% | This work |
| RuO_2_ | benzylamine | 1.4 | 50.1 | NA | NA | This work |
| NiSe | benzylamine | 1.4 | ≈ 140 | NA | ≈ 99% | ^9^ |
| Ni_2_P | benzylamine | 1.5 | 80 | NA | >90% | ^10^ |
| CoSe_2_/Ni−SVs | benzylamine | 1.5 | 120 | NA | NA | ^11^ |
| Mo_0.8_Ni_0.2_N-Ni_3_N | benzylamine | 1.42 | ≈140 | 97.5% | 97.2% | ^12^ |
| Fe-Ni_3_S_2_ | benzylamine | 1.5 | ≈ 85 | NA | ≈95% | ^13^ |
| Co_2_P_4_O_12_ | benzylamine | 1.4 | ≈ 185 | NA | >95% | ^14^ |
| Ru-Ni_2_P | benzylamine | 1.4 | 100 | NA | 96.3% | ^15^ |
| Vo-rich CuO | benzylamine | 1.36 | ≈ 12 | NA | 93.8% | ^16^ |
| W-Ni_2_P | benzylamine | 1.44 | ≈160 | NA | ≈95% | ^17^ |
| Vacancy-rich Ni(OH)_2_ | propylamine | 1.42 | ≈60 | NA | 90.9% | ^18^ |
| S-Ni(OH)_2_ | propylamine | 1.33 | 100 | NA | NA | ^19^ |
| NiO/NC | Cyclohexenylethylamine | 1.4 | ≈18 | NA | ≈99% | ^20^ |

**References**

1. G. Kresse, J. Furthmuller, *Comput. Mater. Sci.* **1996**, *6*, 15.
2. G. Kresse, J. Furthmuller, *Phys. Rev. B* **1996**, *54*, 11169.

3. J. P. Perdew, K. Burke, M. Ernzerhof, *Phys. Rev. Lett.* **1996**, *77*, 3865.

4. P. E. Blöchl, *Phys. Rev. B* **1994**, *50*, 17953−17979.

5. S. Grimme, S. Ehrlich, L. Goerigk, *J. Comput. Chem.* **2011**, *32*, 1456.

6. M. Casas-Cabanas, J. Canales-Vázquez, J. Rodríguez-Carvajal, M. R. Palacín, *J. Am. Chem. Soc.* **2007**, *129*, 5840.

7. S. Dudarev, G. Botton, S. Savrasov, C. Humphreys, A. Sutton, *Phys. Rev. B* **1998**, *57*, 1505.

8. K. Mathew, R. Sundararaman, K. Letchworth-Weaver, T. A. Arias, R. G. Hennig, *J. Chem. Phys.* **2014**, *140*, 084106.

9. Y. Huang, X. Chong, C. Liu, Y. Liang and B. Zhang, *Angew. Chem. Int. Ed.*, **2018**, *57*, 13163−13166.

10. Y. Ding, B.-Q. Miao, S.-N. Li, Y.-C. Jiang, Y.-Y. Liu, H.-C. Yao and Y. Chen, *Appl. Catal. B*, **2020**, *268*, 118393.

11. L. Zeng, W. Chen, Q. Zhang, S. Xu, W. Zhang, F. Lv, Q. Huang, S. Wang, K. Yin, M. Li, Y. Yang, L. Gu and S. Guo, *ACS Catal.*, **2022**, *12*, 11391−11401.

12. Y. Li, Y. Jiao, H. Yan, G. Yang, Y. Liu, C. Tian, A. Wu and H. Fu, *Angew. Chem. Int. Ed.*, **2023**, *62*, e202306640.

13. L. Sun, Z. Zhou, Y. Xie, J. Zheng, X. Pan, L. Li and G. Zhao, *Adv. Funct. Mater.*, **2023**, *33*, 2301884.

14. K. Chen, W. Zhang, Y. Bai, W. Gong, N. Zhang, R. Long and Y. Xiong, *Chin. Chem. Lett.*, **2023**, *34*, 107319.

15. X. Liu, X. He, Z. Fang, S. Gong, D. Xiong, W. Chen, J. Wang and Z. Chen, *Chem. Mater.*, **2024**, *36*, 968−979.

16. X. Yang, E. Wei, Y. Dong, Y. Fan, H. Gao, X. Luo and W. Yang, *Chem. Sci.*, **2024**, *15*, 12580−12588.

17. Z. Tu, X. He, X. Liu, D. Xiong, J. Zuo, D. Wu, J. Wang and Z. Chen, *Chin. J. Catal.*, **2024**, *58*, 146−156.

18. W. Wang, Y. Wang, R. Yang, Q. Wen, Y. Liu, Z. Jiang, H. Li and T. Zhai, *Angew. Chem. Int. Ed.*, **2020**, *59*, 16974−16981.

19. Q. Wen, Y. Lin, Y. Yang, R. Gao, N. Ouyang, D. Ding, Y. Liu and T. Zhai, *ACS Nano*, **2022**, *16*, 9572−9582.

20. X. Pan, L. Sun, Z. Zhou, Y. Xie, J. Zheng, S. Xu, J. Sun, J. Zeng and G. Zhao, *Adv. Energy Mater.*, **2024**, *14*, 2400374.
